# Supplementary material for: ABA-AA: A simple, reversible, and non-toxic anchor-away system for effective nuclear protein depletion
Source: Cell Rep Methods. 2026 Jul 8;6(9):101517. doi: 10.1016/j.crmeth.2026.101517 (PMC13615496; doi:10.1016/j.crmeth.2026.101517)
Supplement: Supplementary file 1 [file mmc1.pdf]

Cell Reports Methods, Volume *6*

Supplemental information

ABA-AA: A simple, reversible, and non-toxic anchor-away system for effective nuclear protein depletion

Sofia Esteban-Serna, Tove Widén, Simon Seliner, Hanne Grosemans, Iseabail Farquhar, Matthew P. Swaffer, and Sander Granneman

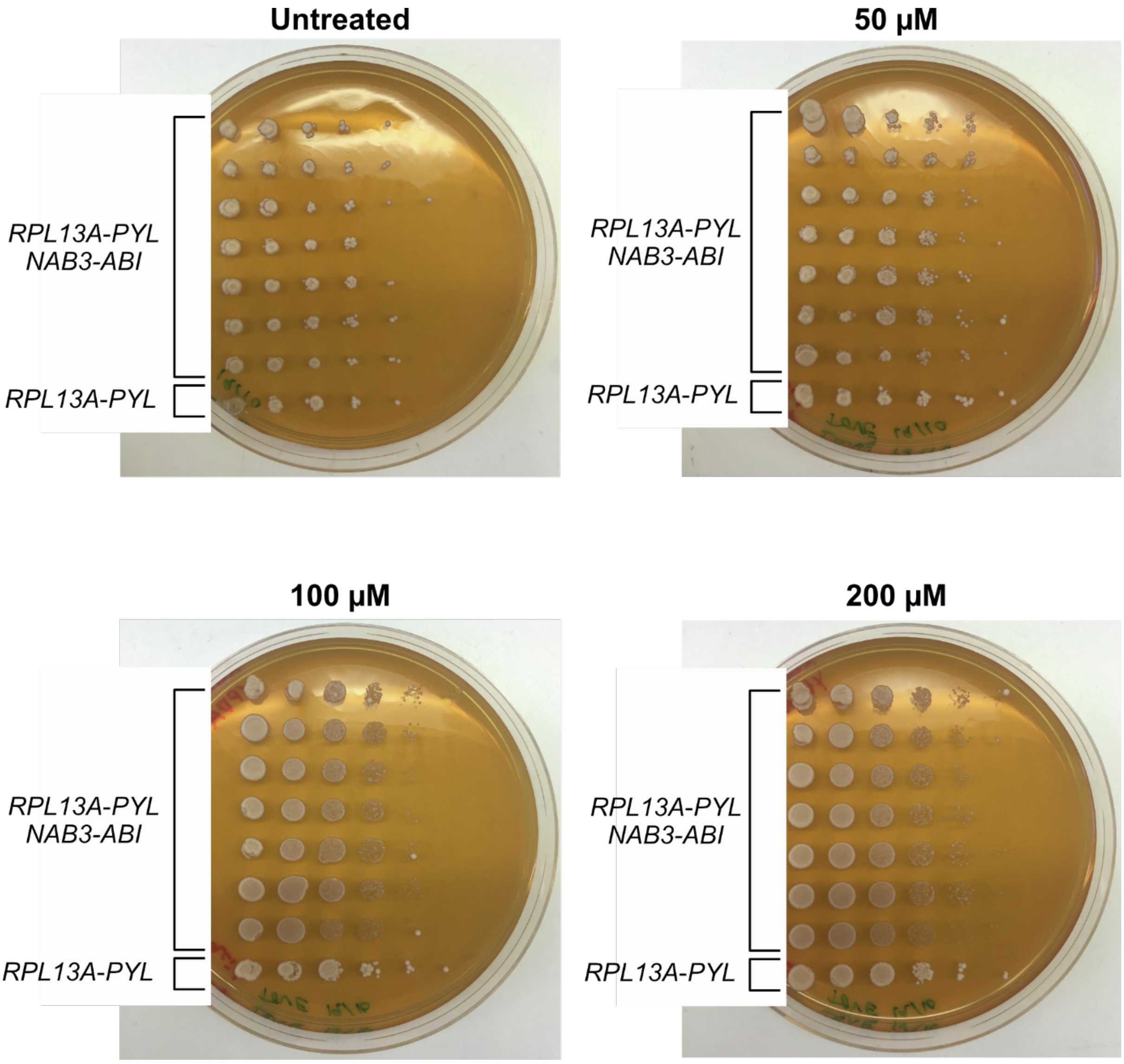

Figure S1. ABA concentrations higher than 200 µM are required to significantly deplete Nab3 from the nucleus using ABA-AA. Related to Figure 2.
Results from representative serial dilution growth analyses of seven *RPL13A-PYL NAB3-ABI* clones and one control strain (*RPL13A-PYL*) on YPD plates without ABA (untreated) or 50 µM, 100 µM and 200 µM ABA.

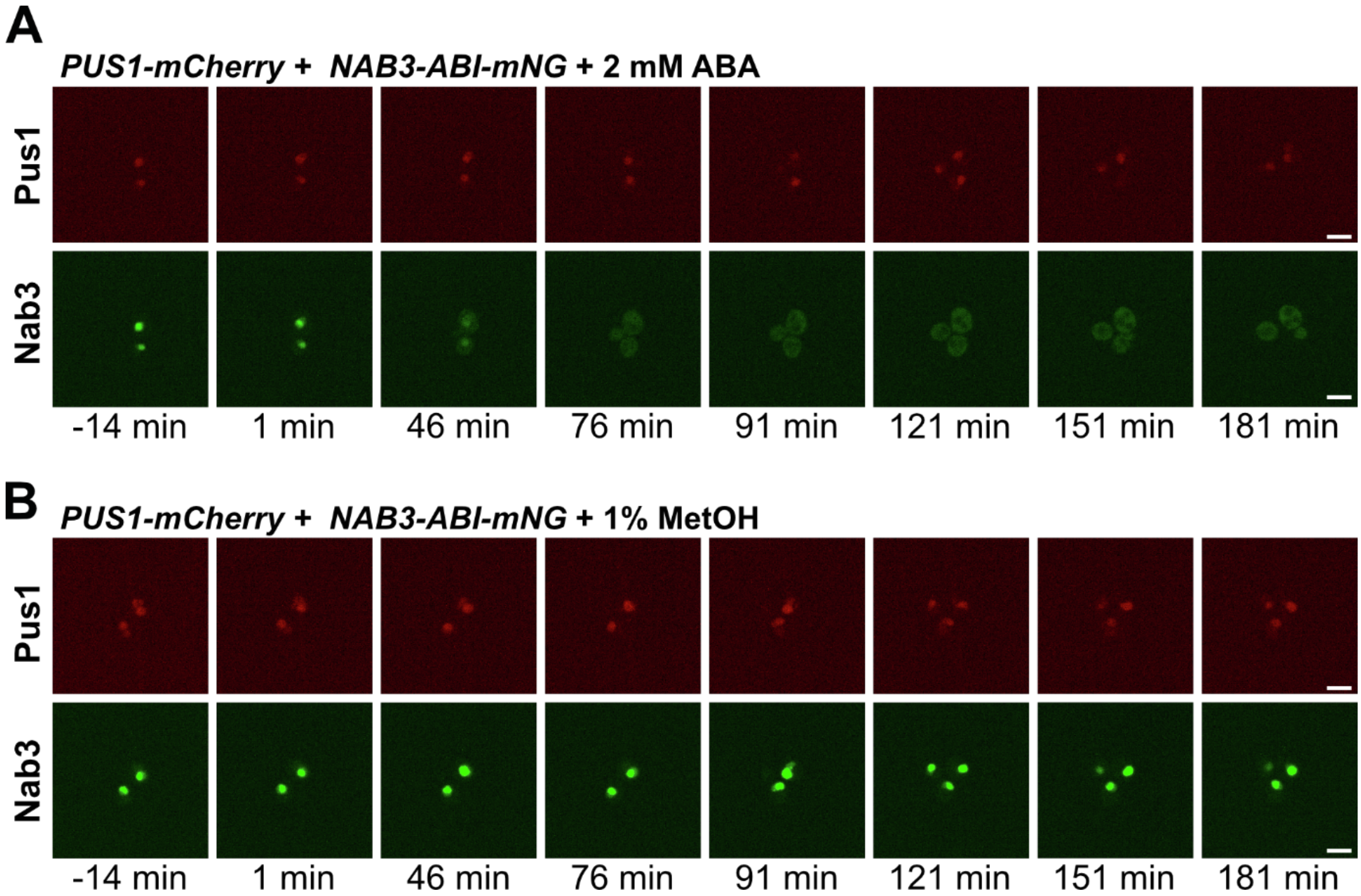

Figure S2. ABA-AA effectively depletes Nab3 from the nucleus. Related to Figure 5. Representative images of cells expressing Nab3-ABI-mNeonGreen. Images were acquired at the indicated time points relative to treatment with 2 mM ABA (**A**) or 1% methanol solvent (**B**). The top row shows the Pus1-mCherry signal, used as a nuclear marker. The bottom row shows the mNeonGreen signal. Quantification of the results is provided in Fig. 5A. White scale bar represents 5 µm.

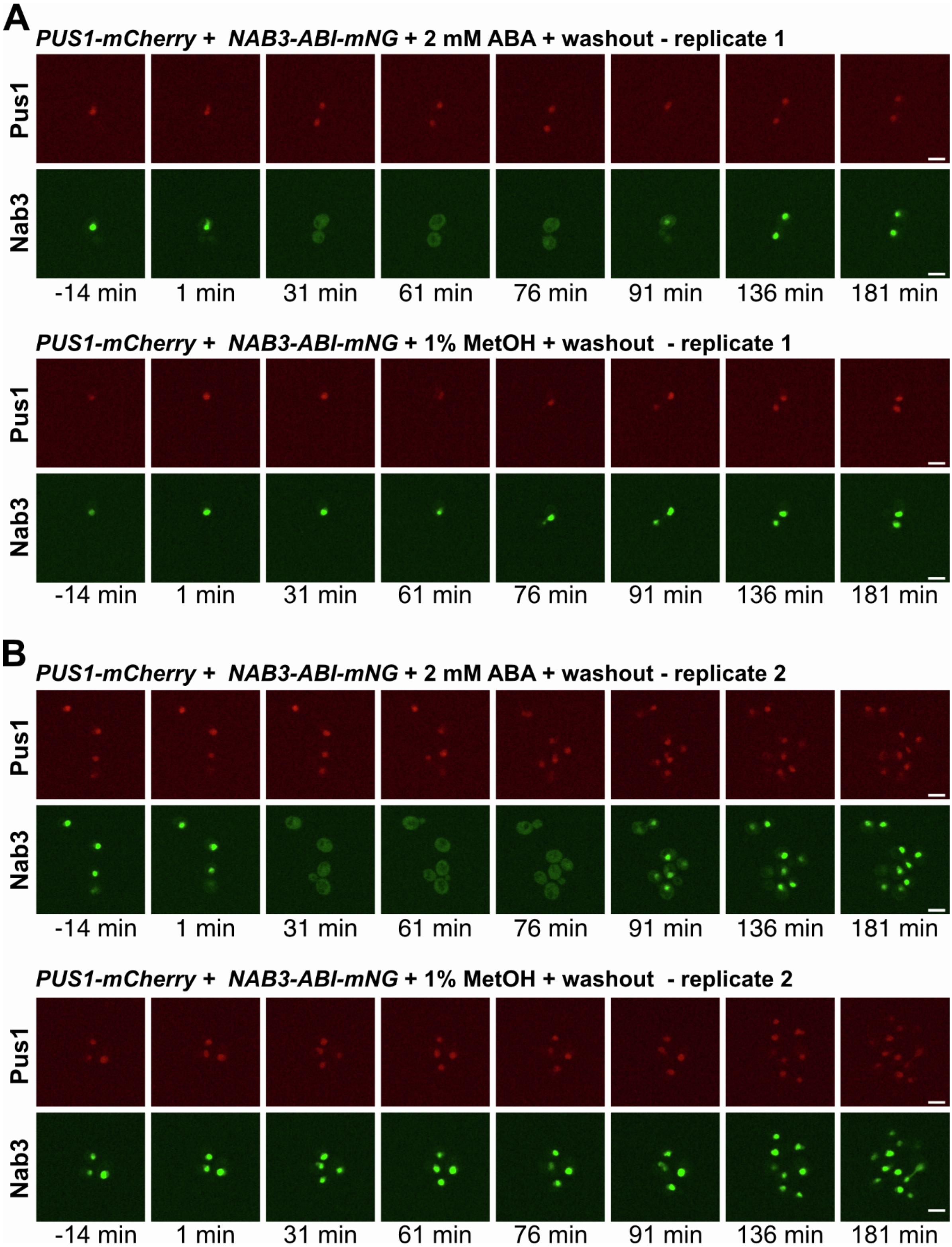

Figure S3. Fully reversible nuclear depletion of Nab3 using ABA-AA. Related to Figure 5. Representative images of cells expressing Nab3-ABI-mNeonGreen. Images were acquired at the indicated time points relative to initial treatment with 2 mM ABA (**A**) or 1% methanol solvent (**B**). ABA and methanol solvent were then washed out with fresh media at 73 minutes. The top row shows the Pus1-mCherry signal, used as a nuclear marker. The bottom row shows the mNeonGreen signal. Quantification of the results is provided in Fig. 5C. White scale bar represents 5 µm.

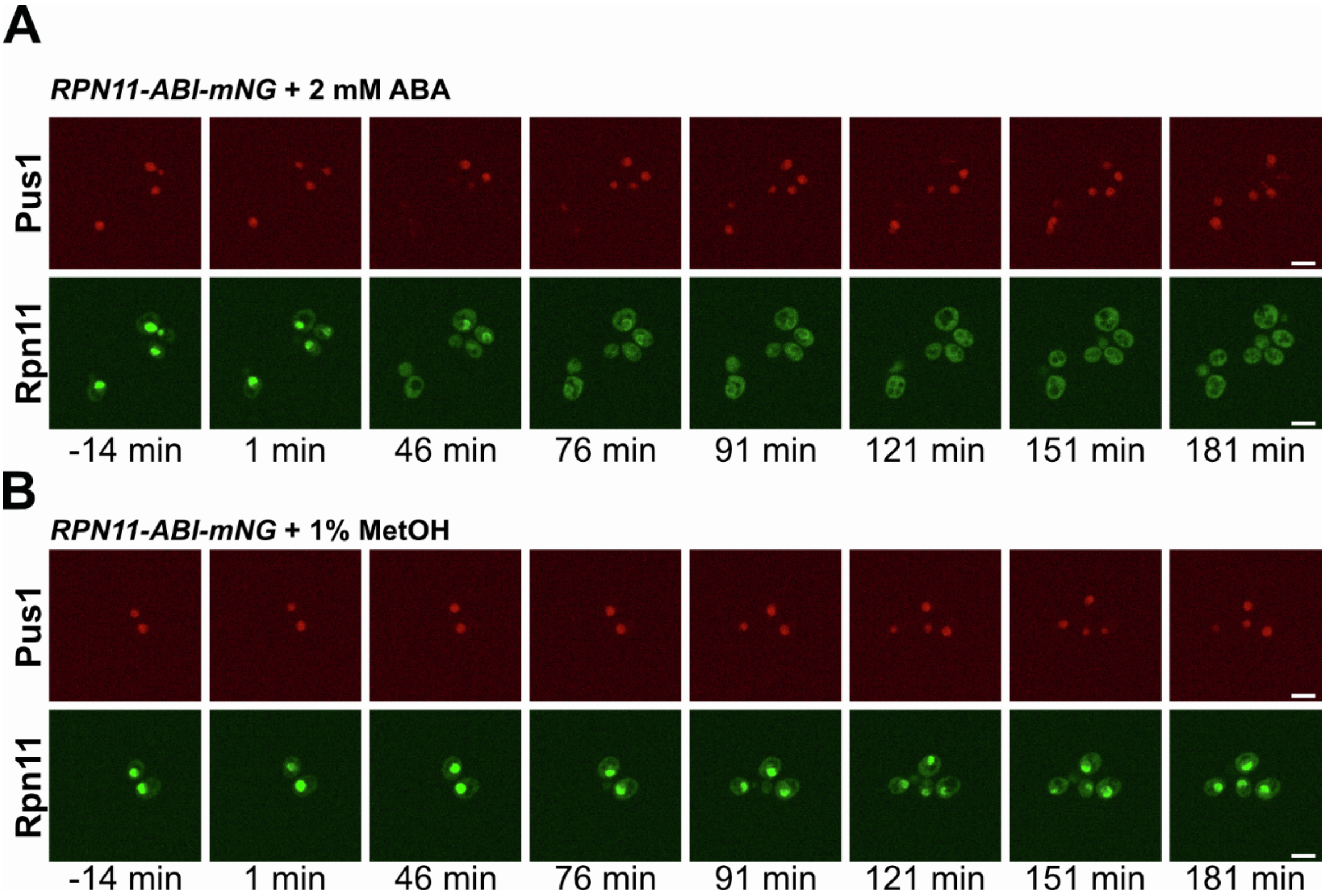

Figure S4. ABA-AA effectively depletes Rpn11 from the nucleus. Related to Figure 5. Representative images of cells expressing Rpn11-ABI-mNeonGreen. Images were acquired at the indicated time points relative to treatment with 2 mM ABA (**A**) or 1% methanol solvent (**B**). The top row shows the Pus1-mCherry signal, used as a nuclear marker. The bottom row shows the mNeonGreen signal. Quantification of the results is provided in Fig. 5B. White scale bar represents 5 µm.

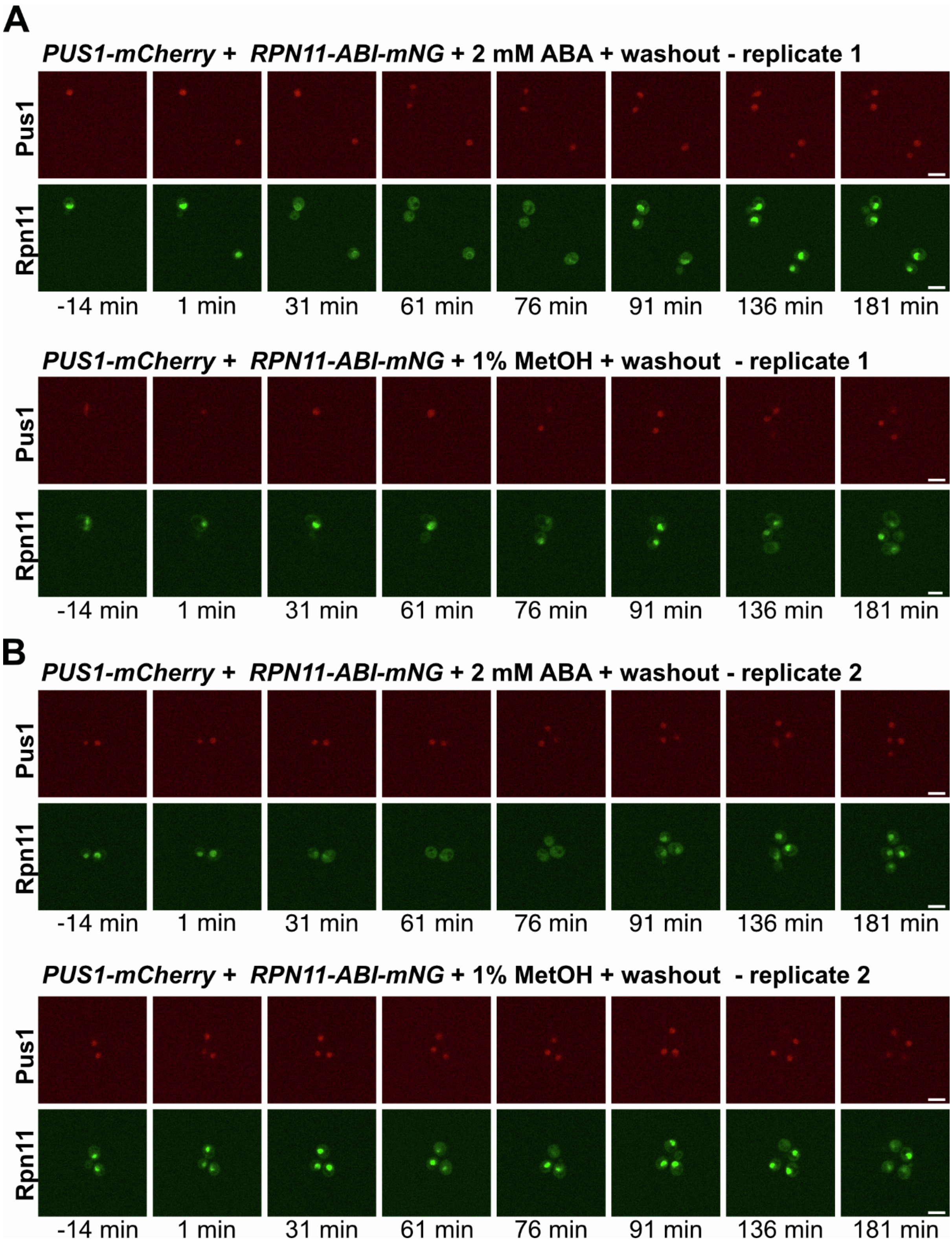

Figure S5. Fully reversible nuclear depletion of Rpn11 using ABA-AA. Related to Figure 5. Representative images of cells expressing Rpn11-ABI-mNeonGreen. Images were acquired at the indicated time points relative to initial treatment with 2 mM ABA (**A**) or 1% methanol solvent (**B**). ABA and methanol were then washed out with fresh media at 73 minutes. The top row shows the Pus1-mCherry signal, used as a nuclear marker. The bottom row shows the mNeonGreen signal. Quantification of the results is provided in Fig. 5D. White scale bar represents 5 μm.
