# Supplementary material for: ABA-AA: A simple, reversible, and non-toxic anchor-away system for effective nuclear protein depletion
Source: Cell Rep Methods. 2026 Jul 8;6(9):101517. doi: 10.1016/j.crmeth.2026.101517 (PMC13615496; doi:10.1016/j.crmeth.2026.101517)
Supplement: Supplementary file 2 [file mmc7.pdf]

Cell Reports Methods

Article

# ABA-AA: A simple, reversible, and non-toxic anchor-away system for effective nuclear protein depletion

## Graphical abstract

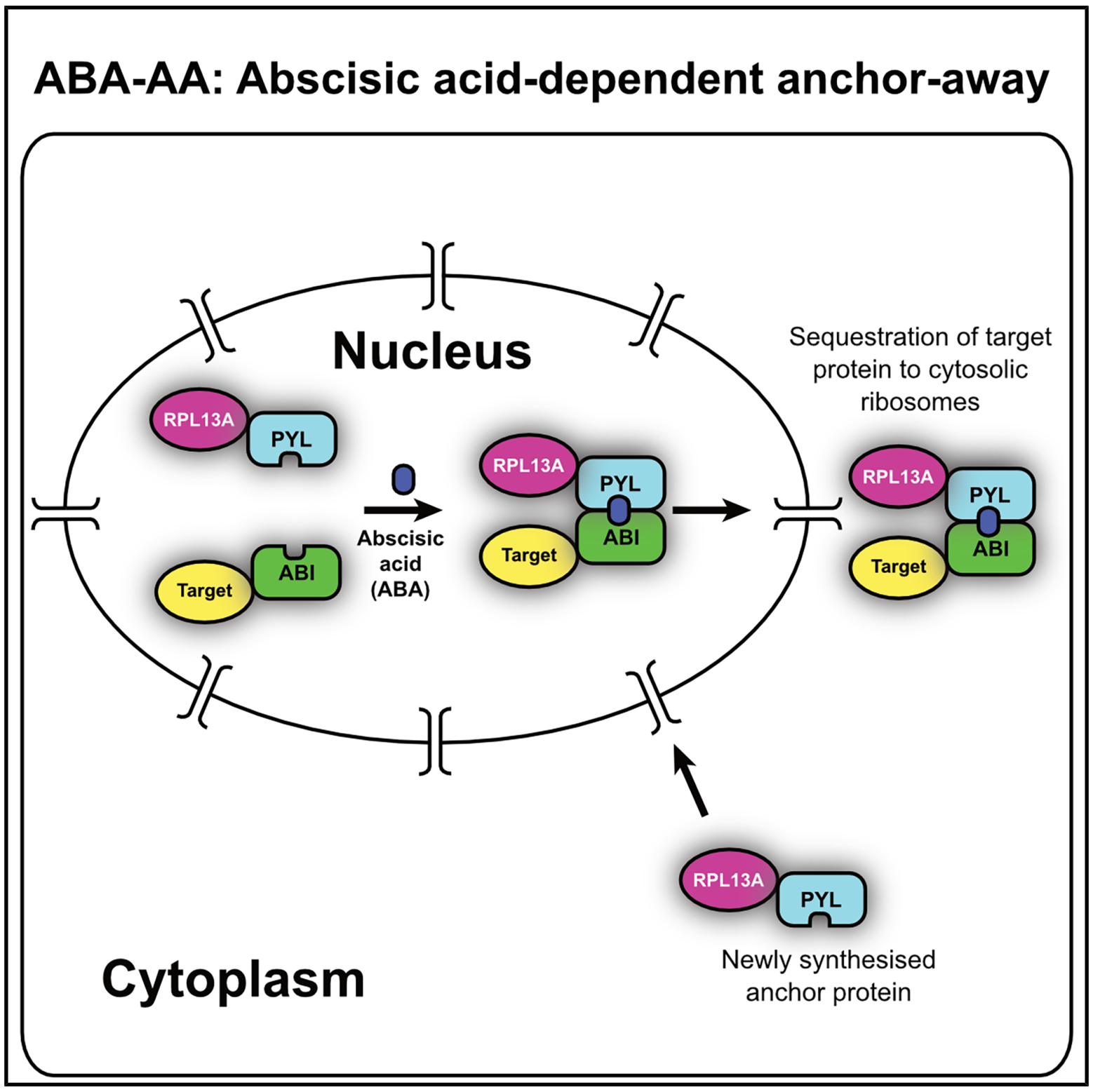


## Authors

Sofia Esteban-Serna, Tove Widén, Simon Seliner, Hanne Grosemans, Iseabail Farquhar, Matthew P. Swaffer, Sander Granneman

## Correspondence

sander.granneman@ed.ac.uk

## In brief

Esteban-Serna et al. describe an anchor-away system that uses the plant hormone abscisic acid (ABA) to rapidly deplete proteins from the nucleus in budding yeast. Unlike existing approaches, the system is inexpensive, non-toxic, fully reversible, and works across different genetic backgrounds, making it broadly applicable for studying dynamic cellular processes.

## Highlights

- ABA-AA enables rapid and fully reversible nuclear protein depletion in yeast
- ABA is non-toxic and does not significantly alter yeast gene expression
- ABA-AA can effectively deplete highly abundant nuclear proteins
- ABA-AA is applicable across diverse genetic backgrounds and eukaryotic model systems

Esteban-Serna et al., 2026, Cell Reports Methods *6*, 101517
September 21, 2026 © 2026 The Authors. Published by Elsevier Inc.
https://doi.org/10.1016/j.crmeth.2026.101517

CellPress

Cell Reports Methods

CellPress
OPEN ACCESS

Article

# ABA-AA: A simple, reversible, and non-toxic anchor-away system for effective nuclear protein depletion

Sofia Esteban-Serna,[1,3,4] Tove Widén,[2,4] Simon Seliner,[3] Hanne Grosemans,[1] Iseabail Farquhar,[1] Matthew P. Swaffer,[3] and Sander Granneman[1,3,5,*]

[1]Centre for Engineering Biology, Institute for Quantitative Biology, Biochemistry and Biotechnology, University of Edinburgh, Edinburgh EH9 3BF, UK

[2]Department of Life Sciences, Chalmers University of Technology, 412 96 Gothenburg, Sweden

[3]Centre for Cell Biology, Institute of Cell Biology, University of Edinburgh, Edinburgh EH9 3BF, UK

[4]These authors contributed equally

[5]Lead contact

*Correspondence: sander.granneman@ed.ac.uk

https://doi.org/10.1016/j.crmeth.2026.101517

**MOTIVATION** Existing tools for conditional nuclear protein depletion carry liabilities that limit their utility in certain experimental contexts. Promoter-based strategies require alterations to nutrient conditions and extended incubation periods, while some degron-based systems demand careful titration to avoid basal target degradation. The widely used rapamycin-dependent AA system is fast-acting in yeast but costly: the drug inhibits TOR signaling and therefore requires a specialized genetic background that compromises the heat-shock response. This renders it incompatible with studies of stress-induced transcriptional reprogramming. Here, we describe an AA system based on abscisic acid (ABA)-dependent dimerization that sidesteps these drawbacks and is also fully reversible, offering a broadly applicable, inexpensive, non-toxic alternative for nuclear protein depletion in eukaryotes.

## SUMMARY

The anchor-away (AA) technique enables rapid depletion of nuclear proteins by tethering them to cytoplasmic anchors through rapamycin-induced heterodimerization. In *Saccharomyces cerevisiae*, this system is restricted to rapamycin-resistant strains, as the drug inhibits TOR signaling and hinders the heat-shock response, precluding its application in stress-related studies. Moreover, this AA method is not rapidly reversible, limiting studies that require transient perturbation and functional restoration. To overcome these constraints, we developed an alternative AA system that uses the plant hormone abscisic acid (ABA) to induce conditional association of the target to its cytoplasmic anchor. Gene expression and microscopy analyses demonstrate that the ABA-AA system enables rapid and fully reversible depletion of highly abundant nuclear proteins. Unlike rapamycin, ABA is non-toxic, does not cause major gene expression changes, and is suitable for diverse genetic backgrounds. The ABA-AA system, therefore, provides a broadly applicable alternative for nuclear protein depletion across eukaryotic systems.

## INTRODUCTION

Common approaches to characterizing protein function in yeast include permanently knocking out the gene encoding it or generating a conditional allele (e.g., a temperature-sensitive mutant). However, essential genes cannot be deleted or inactivated, and generating temperature-sensitive mutants can be labor-intensive, often leading to slow loss of function of the protein of interest. Moreover, in the latter case, the required temperature increases can trigger heat stress responses, which can overshadow the adaptive response caused by the deactivation of the protein of interest.

A popular alternative strategy to conditional gene knockouts is genetic depletion, which can be mediated by promoters or riboswitches. In most instances, endogenous promoters are replaced with inducible ones. Typical conditional promoters include the native *pGAL* and *pMET*, which are turned off by glucose and methionine, respectively,[1,2] or heterologous promoters that are repressed in the presence of doxycycline/tetracycline.[3–5] Similarly, riboswitches inserted into the 5′ untranslated region


This is an open access article under the CC BY license (http://creativecommons.org/licenses/by/4.0/).

(UTR) of the target gene can, upon ligand binding, block ribosome scanning and thus modulate translation initiation of the gene product in a controlled manner.[6–8] The disadvantages of these conditional depletion systems are that they often involve altering culture nutrient conditions and long incubation periods to achieve sufficient depletion of the target protein.

In contrast, engineered chemically induced dimerization (CID) methodologies can often achieve significant and rapid depletion of the target protein, often in less than 30 min, thereby minimizing noise introduced by secondary responses. CID systems employ inducers, which are small membrane-permeable molecules that promote stable interactions between two distinct protein domains. Such is the mode of action underlying the two most broadly used CID variants, namely the auxin-inducible degron (AID) and the anchor-away (AA) methods[9–15] (Figure 1A). In the AID system, the target is tagged with an AID epitope that binds the rice protein TIR1 in the presence of auxin (Figure 1A). In turn, TIR1 recruits ubiquitin ligases to polyubiquitinate the protein of interest, marking it for proteasomal degradation (Figure 1A).

The AA technique relies on ligand-induced export of the target protein to the cytoplasm, where it remains sequestered on ribosomes or membrane-bound factors.[14] This procedure leverages the ability of the human 12 kDa FK506 binding protein (FKBP12) and the 11 kDa FKBP12-rapamycin-binding (FRB) domain of human mTOR to form a stable heterodimer in the presence of rapamycin[16] (Figure 1B). In the original version of the protocol, the target protein is marked with the FRB domain, and the FKBP12 domain is genetically fused to the ribosomal protein Rpl13A,[17] allowing the latter to act as a highly abundant cytoplasmic anchor (Figure 1B). Following its translation, Rpl13A is imported into the nucleus, where it is incorporated into 60S pre-ribosomal particles (Figure 1B). Bound to almost fully mature 60S particles, Rpl13A is exported back to the cytoplasm, where it then contributes to protein biosynthesis (Figure 1B). Therefore, in the presence of rapamycin, the target-FRB protein is dragged out of the nucleus and sequestered to translating ribosomes in the cytoplasm (Figure 1B).

Despite proving tremendously powerful, each of these CID methods has disadvantages. The original AID system was hindered by the cytotoxicity of auxin. The hormone can also undergo photodecomposition into harmful derivatives[18] that, if accumulated, cause growth defects. Additionally, the method displayed basal or "leaky" degradation of the target protein as TIR1 weakly associates with the tagged target even in the absence of auxin, leading to unintended proteasomal degradation.[19] These problems have largely been alleviated by substituting auxin with indole-free analogs[18] that are more stable and less toxic or by the AID2 methodology, which uses bump-and-hole engineering to prevent basal proteolysis and vastly reduces the ligand concentration required to induce target depletion.[20] Regardless, the expression of the TIR1 protein still needs to be carefully tuned, as too high levels can induce uncontrolled breakdown of the target protein even in the absence of auxin.[15,20,21]

The primary liability of the rapamycin-dependent AA system is its reliance on rapamycin. In addition to being expensive, this drug can bind to the yeast homolog of FKBP12 (Fpr1) to form an inhibitory complex that blocks the kinase activity of Tor1,

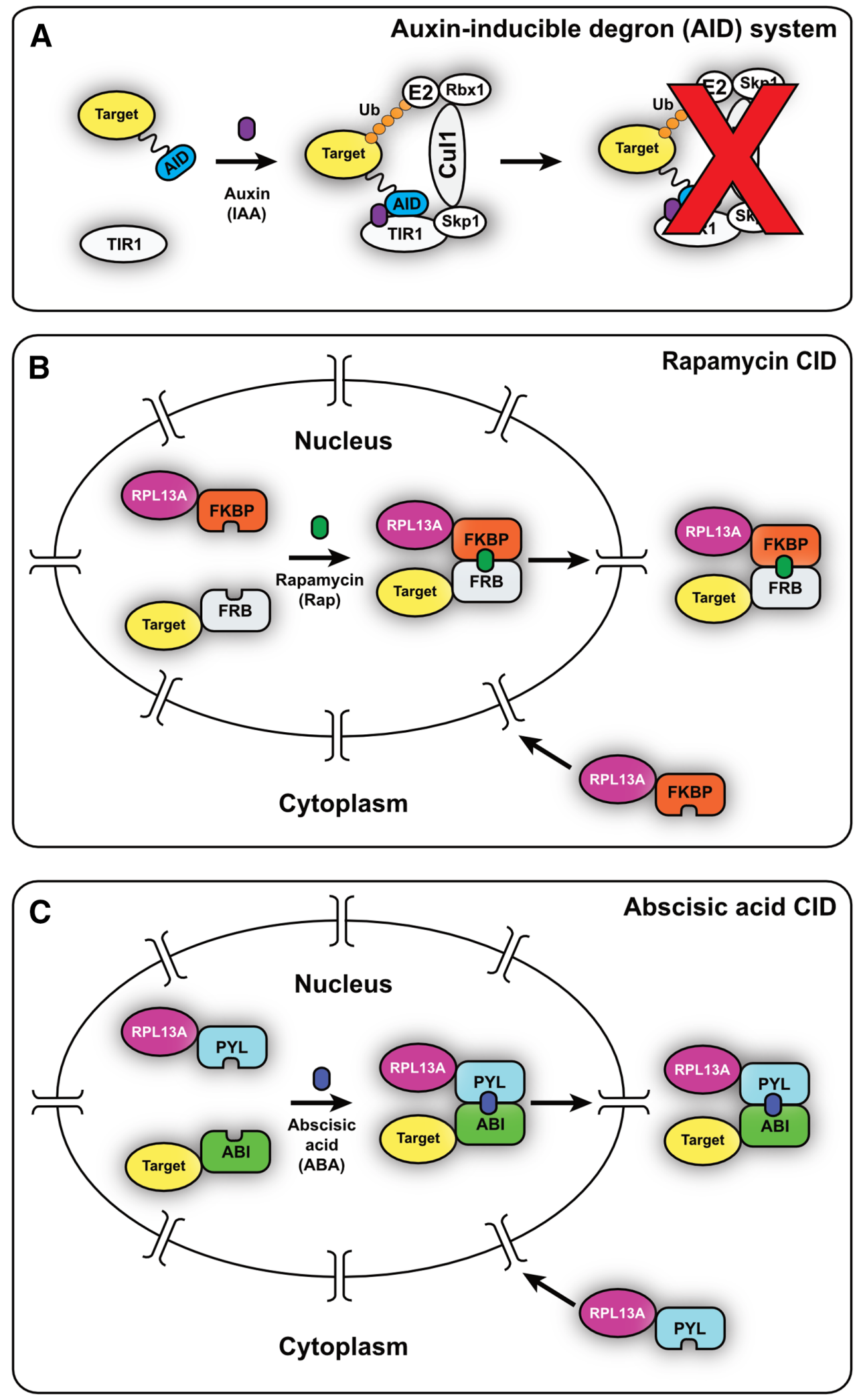


**Figure 1. Chemically induced dimerization approaches for conditional depletion of nuclear proteins**

(A) The auxin-inducible degron (AID) system requires the AID epitope to be fused to the protein of interest. Upon auxin addition, the TIR1 auxin receptor binds the AID degron tag, recruiting the Skp, Cullin F-box (SCF) E3 ubiquitin ligase complex (Skp1, Cul1, and Rpx1), which polyubiquitylates the AID tag and promotes proteasomal degradation of the target protein.

(B) Rapamycin-dependent anchor-away (AA) technique. Newly synthesized Rpl13A-FKBP anchor protein is rapidly imported into the nucleus and incorporated into pre-ribosomal particles. In the presence of rapamycin, the anchor forms a tight complex with FRB-fused target proteins and, when associated with pre-60S complexes, drags the target protein into the cytoplasm, where it is sequestered on translating ribosomes.

(C) The ABA-AA system fuses the PYL domain to the Rpl13A anchor. Here, abscisic acid (ABA) acts as chemical inducer triggering dimerization of the PYL-tagged Rpl13A to the ABI-tagged target protein.

which regulates cell growth, metabolism, and nutrient responses as a key component of the TOR signaling. To circumvent this problem, the AA technique must be applied to rapamycin-resistant strains, such as the HHY168 W303 strain that encodes a mutant version of *TOR1* (*tor1-1*) and lacks *FPR1* (Δ*fpr1*). Being the most abundant FK506-binding protein in yeast,[22] Fpr1 was

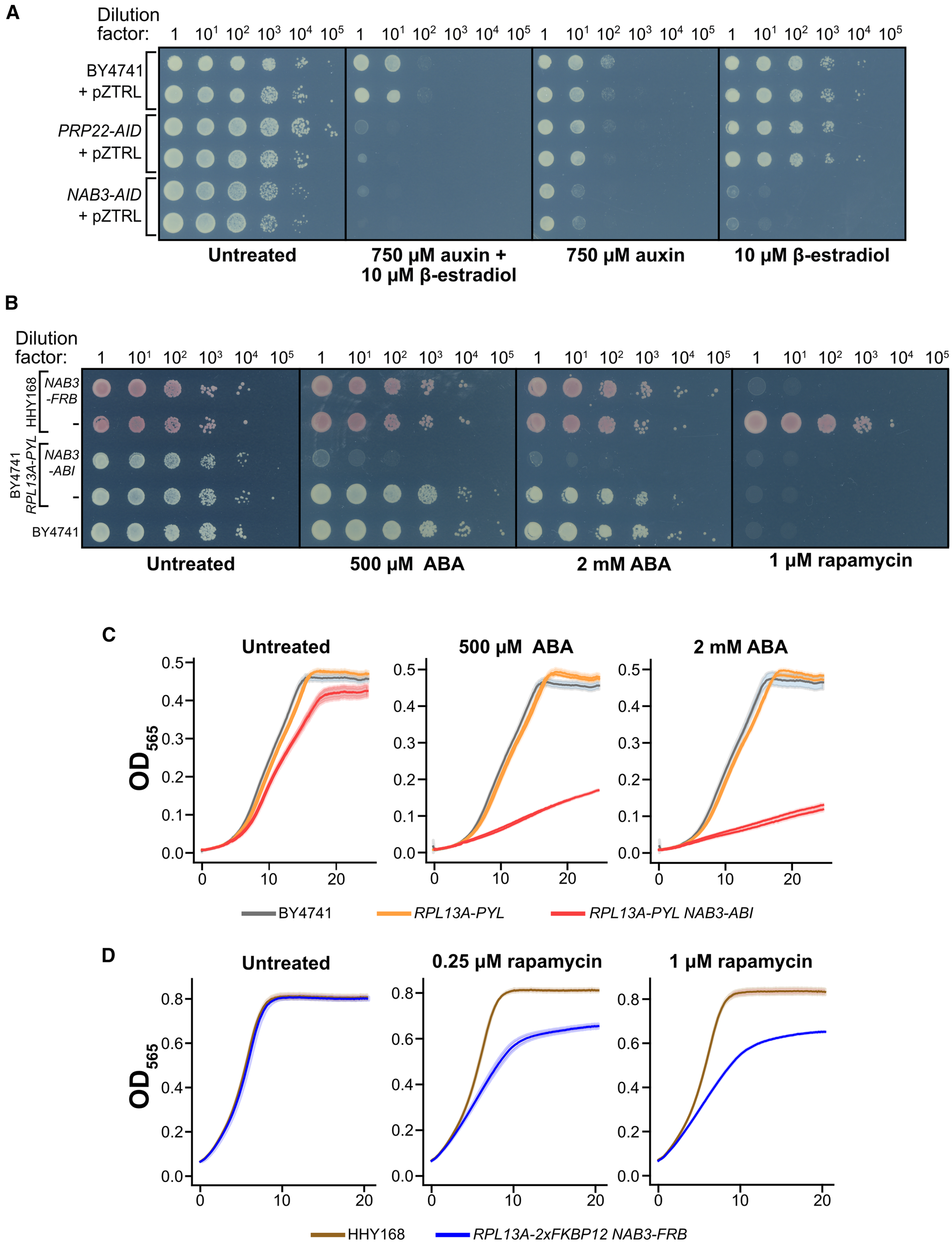


**Figure 2. ABA is a non-toxic inducer that drives controlled depletion of Nab3 as efficiently as reported AID or rapamycin-dependent systems**

(A) Spot test analyses of the AID system. *NAB3-AID* and *PRP22-AID* carried a pZTRL plasmid encoding the TIR1 auxin receptor under a β-estradiol-inducible promoter. *PRP22-AID* and BY4741 served as positive and negative controls, respectively. Serial dilutions were spotted onto plates containing minimal medium supplemented with glucose with the indicated doses of auxin and/or β-estradiol and incubated at 30°C for 48 h.

(B) Spot test analysis comparing ABA and rapamycin-dependent AA systems. ABA selectively inhibited growth in *NAB3-ABI* while remaining innocuous to *NAB3-FRB* and parental strains. Rapamycin impaired colony formation in all strains except HHY168, a rapamycin-resistant W303 strain lacking *FPR1* and carrying

*(legend continued on next page)*

also removed in HHY168 to ensure that it would not compete with the FRB-tagged targets for the available FKBP12-fused anchors.[23] Apart from being technically challenging to implement in different background strains, this required genetic background (*tor1-1* and *fpr1Δ*) also diminishes the cell's capacity to activate the heat-shock response.[24]

We have been investigating the role of the Nrd1-Nab3-Sen1 (NNS) transcriptional termination complex in regulating rapid adaptive responses in yeast upon changes in nutrient availability.[25,26] Accordingly, the rapamycin AA strategy is not ideally suited to investigating these proteins, which orchestrate transcriptional reprogramming of its targets precisely under these stress conditions.[26–30]

To address some of the problems associated with this AA system, we have modified a CID system that exploits the plant phytohormone abscisic acid (ABA) to trigger dimer formation between a yeast target protein and its cytoplasmic anchor. Prior to our work, this CID had been successfully implemented in mammalian systems[31] (Figure 1C). In plants, ABA docks into the PYL domain of the RCAR, a receptor of the ABA signaling pathway, enabling the latter to associate with the ABI domain of the ABI1 protein phosphatase.[32] This ABA-dependent CID event has been harnessed to build a synthetic spindle checkpoint in fission yeast.[33] Inspired by this study, we recognized the potential of ABA in an AA system: it is inexpensive, non-toxic, and, since it engages in relatively weak interactions with PYL, its effect can be readily reversed by simply washing out the hormone[31,33,34] (Figure 1C).

Here, we demonstrate that ABA-dependent CID can be effectively employed as an AA system to deplete nuclear proteins in *Saccharomyces cerevisiae*. We show that ABA-AA rapidly and reversibly sequesters the abundant nuclear protein Nab3 in the cytoplasm and can deplete other highly abundant proteins, including the proteasome component Rpn11. Compared with the rapamycin-dependent variant, the ABA-AA requires longer incubation times and higher doses of the ABA ligand to deplete the target protein and elicit the accompanying phenotypic effects. Nevertheless, we demonstrate that, contrary to other ligands used, ABA is innocuous, as it does not significantly impair cellular growth or dramatically alter gene expression in exponentially growing cells, even at very high concentrations. Moreover, we show that, unlike existing AA approaches, it is also fully reversible. This makes ABA-AA ideal for studying dynamic processes. Requiring the generation of only two fusion proteins, the ABA-AA system is readily applicable to diverse genetic backgrounds, environmental conditions, and other eukaryotic organisms.

## RESULTS AND DISCUSSION

### ABA-AA recapitulates Nab3 depletion without toxicity in *S. cerevisiae*

We, as well as others, have previously employed the rapamycin AA system to successfully deplete a variety of nuclear RNA surveillance factors, demonstrating the effectiveness of this approach.[26,35–38] However, as it relies on the *tor1-1* and *fpr1Δ* mutations, this procedure may affect the TOR signaling pathway, which is intimately linked to nutrient sensing.[39,40] We reasoned that introducing disturbances in that metabolic route was not ideal while examining the function of proteins in adaptation to nutrient starvation. One such factor is Nab3, an essential RNA-binding protein involved in transcription termination that orchestrates transcriptional reprogramming during glucose or nucleotide shortage.

We, therefore, sought an alternative strategy for depleting Nab3 from the nucleus without interfering with TOR kinase activity. We first tested an optimized yeast auxin-induced degron (AID) system[15] (Figure 1A), which represented the state-of-the-art at the time this project started. This procedure involves fusing the AID domain to the Nab3 C terminus and transforming the strain with a plasmid encoding a β-estradiol-inducible version of *TIR1* (pZTRL). To test whether this procedure triggers the growth defects associated with loss of Nab3, we exposed the resulting Nab3-AID + pZTRL strain to different growth conditions: (1) the absence of any ligands (Figure 2A; "untreated"); (2) a medium containing a mix of auxin and β-estradiol, wherein *TIR1* would be expressed and able to induce degradation of the AID-labeled target; (3) plates containing β-estradiol to induce *TIR1* expression; and (4) auxin alone, which by itself can moderately impair cell growth (Figure 2A). To confirm these expectations, we monitored the growth of a strain in which the splicing factor Prp22 had been previously depleted with the same AID system (Figure 2A).[15] Conversely, the BY4741 reference strain was used as a negative control for tolerance to auxin and heterologous *TIR1* expression (Figure 2A).

As anticipated,[15] the AID system efficiently degraded Prp22-AID in an auxin- and *TIR1*-dependent manner (Figure 2A). However, β-estradiol-induced *TIR1* expression alone caused substantial growth defects in the Nab3-AID strain, indicating auxin-independent degradation (Figure 2A). This finding reemphasizes the importance of carefully fine-tuning *TIR1* induction and production.[15,21] Notably, tuning *TIR1* expression remains a caveat even in improved systems like AID2, where the auxin-ligand-related drawbacks have almost been eliminated.[20] Addition of auxin alone caused significant growth defects, and this was slightly exacerbated in Nab3-AID (Figure 2A). We propose that, combined with the administration of auxin, leaky expression of *TIR1* from the pZTRL plasmid resulted in uncontrolled proteolysis of Nab3-AID.

We, therefore, aimed to develop a conditional nuclear depletion system that was simpler and had fewer toxicity issues. We evaluated an abscisic acid (ABA)-based CID system. ABA is innocuous to cultured mammalian cells,[31] inexpensive, chemically stable, and has been applied as a dimerization inducer in other yeasts.[33,34] To test whether ABA was indeed harmless to *S. cerevisiae*, we monitored the growth of BY4741 in media containing high concentrations of the hormone (i.e., 500 μM and

*tor1-1*, which served as the parental control for *NAB3-FRB*. BY4741 was included as a negative control. Serial dilutions were spotted onto minimal glucose medium with the indicated dose of ABA or rapamycin.

(C and D) Time-resolved optical density measurements depicting the mean (line) and the standard deviation (shading) of three technical repeats. Two independent clones were examined for each ABA-dependent AA strain.

2 mM, 2 and 8 times more than the tested concentration in fission yeast, respectively[33]; Figures 2B and 2C). Our assays show that even millimolar concentrations of ABA did not noticeably impair cell growth (Figures 2B and 2C).

Next, we assembled the genetic parts needed for a functional ABA-AA system. We constructed a strain expressing a hemagglutinin (HA)-tagged PYL domain (*PYL-3XHA*, 26 kDa) fused to the ribosomal protein Rpl13A (*RPL13A-PYL*). Next, in the *RPL13A-PYL* strain, we then C-terminally fused the FLAG-tagged ABI domain (*ABI-3xFLAG*, 37.4 kDa) to Nab3 (*NAB3-ABI*). We also generated a strain with reversed domain architecture (*RPL13A-ABI* and *NAB3-PYL*). However, Rpl13A-ABI was non-functional as an anchor. We speculate this could be due to the ABA-ABI-PYL interaction geometry or different epitope sizes (25 kDa vs. 37.4 kDa).

Because the ABI and PYL domains comprised relatively large epitopes, we tested whether the target and anchor proteins remained fully functional. Even though *RPL13A* is a non-essential gene, tagging it with the 26 kDa PYL-3HA domain could affect the activity of Rpl13A and inhibit translation to some extent. Moreover, even mild deficiencies of Nab3 termination can severely disrupt cellular physiology.[25] Introducing the *PYL-3xHA* tag downstream of Rpl13A did not significantly alter the growth of *RPL13A-PYL* compared with the BY4741 reference on agar and in liquid cultures (Figures 2B and 2C). However, integrating the ABI domain to the C terminus of Nab3 modestly reduced growth (*RPL13A-PYL-3xHA NAB3-ABI-3xFLAG*) compared with the BY4741 parental strain (Figure 2C; "untreated"). Although such defect is not observed in the *NAB3-FRB* (*RPL13A-2xFKBP12 NAB3-FRB*) strain, some C-terminal fusions are known to slightly reduce Nab3 function.[30] Indeed, the ABI epitope is approximately three times the molecular weight of the FRB domain (37.4 kDa vs. 11 kDa). It is, therefore, not surprising that a larger tag would partially compromise the physiological role of Nab3. This is an important practical consideration when employing the ABA-AA system for other nuclear proteins. Nevertheless, the fact that this growth defect was not immediately apparent from spot-test analyses underscores the mildness of the defect (Figure 2B; "Untreated").

We conclude that the genomic integrations required for our ABA-AA system largely preserved the function of the target and anchor proteins.

We then assessed whether fusing *ABI* and *PYL* to the target (i.e., Nab3) and the anchor (i.e., Rpl13A) allowed us to reproduce the growth defects associated with Nab3 loss in the presence of rapamycin.[30,37,41] We first spotted serially diluted cultures onto solid medium containing a range of ABA concentrations. While 250 μM ABA is sufficient to induce protein relocalization in fission yeast and murine cells,[31,33] spot assays did not reveal a pronounced growth defect in the *NAB3-ABI* strain at concentrations up to 200 μM (Figure S1). Given that Nab3 is a highly abundant protein (Table 1), higher ABA doses may be required for effective depletion. We, therefore, also tested concentrations of 500 μM and 2 mM ABA and found that 500 μM ABA already achieved growth defects (Figures 2B and 2C) comparable to those observed in *NAB3-FRB* treated with 1 μM rapamycin (Figures 2B–2D). We note, however, that growth assays in liquid and solid media showed discrepancies in the severity of the growth defects caused by Nab3 relocation to the cytoplasm in both strains. Spot-test assays pointed toward a greater effect of rapamycin in the *NAB3-FRB* strain than that of *ABA* in the *NAB3-ABI* strain, an effect that becomes particularly apparent at a dilution factor of $10^3$ (Figure 2B). Microplate reader growth assays, however, revealed a more pronounced impact of Nab3 nuclear depletion in the *NAB3-ABI* strain than in its *NAB3-FRB* counterpart (Figures 2B and 2C). This likely reflects three confounding factors. First, growth is an integrative readout that reflects the combined impact of Nab3 loss across all its targets, meaning that even a relatively modest degree of nuclear depletion may be sufficient to substantially impair growth. Second, the *tor1-1* and *Δfpr1* mutations required for rapamycin sensitivity compromise TOR signaling independently of Nab3 depletion, potentially dampening the additional growth defect in the *NAB3-FRB* strain. Finally, this could also be owing to changes in the non-linearity of $OD_{565}$ and cell counts in both strains; W303, the reference strain from which HHY168 is derived, is larger, has a lower overall protein concentration, and has larger vacuoles than BY4741.[42]

**Table 1. Median molecules per cell according to the unified protein abundance dataset generated by Ho et al.**

| Protein | Function | Median abundance (molecules/cell) |
|---|---|---|
| Prp22 | pre-mRNA splicing | 1,099 ± 570 |
| Nab3 | transcription termination | 5,830 ± 2,339 |
| Rpn11 | proteasome component | 16,400 ± 4,500 |
| Rpl13A | ribosomal protein | 51,290 ± 37,073 |

The dataset was generated by Ho et al.[43]

Importantly, the ABA-induced growth defect persisted for 24 h (Figure 2C), confirming that ABA remains stable in liquid medium at 30°C. Together, these results demonstrate that ABA is an innocuous, inexpensive, and stable dimerization agent capable of recapitulating the Nab3 depletion phenotypes previously achieved with the rapamycin-driven AA.

## ABA only marginally impacts gene expression in *S. cerevisiae*

Although 500 μM and 2 mM of ABA did not noticeably affect cell growth, we tested whether it altered gene expression by comparing transcriptomes of BY4741 cultures grown in minimal medium with 1% methanol solvent (control), 500 μM ABA, or 2 mM ABA. After 5 h of exponential growth, cells were harvested for RNA sequencing. The resulting data were processed using the pyCRAC pipeline,[44] and a statistical comparison between treated samples and the methanol control was performed using DESeq2[45] (Figure 3; Table S1).

Gene read counts across the three replicates were highly correlated (Figure 3A), suggesting that the independent experiments generated reproducible results. Likewise, comparison of mean gene read coverage in treated and untreated samples showed that treating cells with ABA or methanol did not have major impacts on gene expression (Figures 3B and 3C). However, ABA treatment did significantly increase expression of several mitochondrial ribosomal RNAs (i.e., 21S rRNA and 15S rRNA),

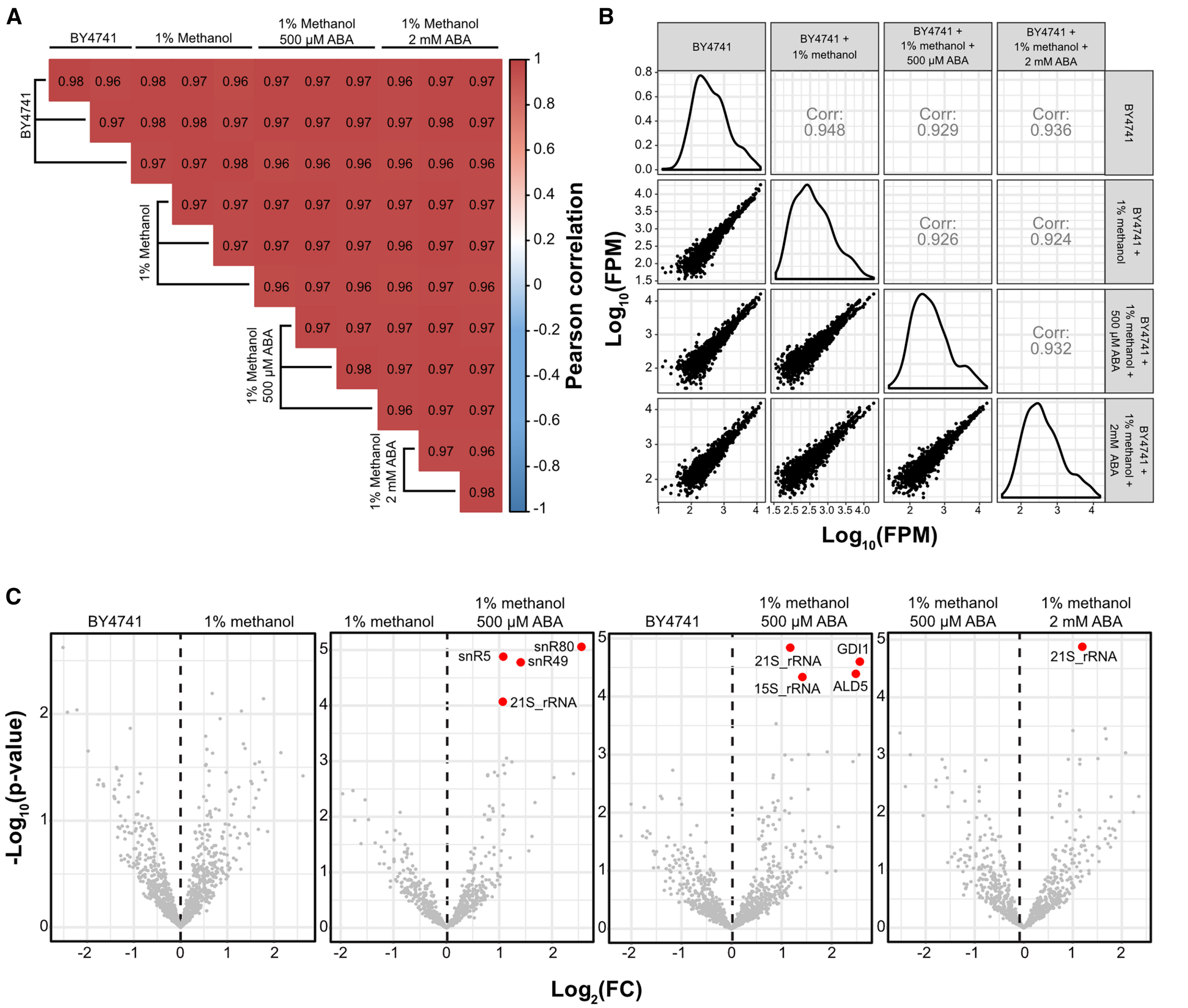


**Figure 3. ABA treatment modestly alters gene expression profiles in *Saccharomyces cerevisiae***

(A) Pearson correlation coefficients between the $n = 3$ independent biological replicates conducted under each condition (i.e., untreated BY4741, 1% methanol, 500 µM ABA, and 2 mM ABA).

(B) Comparison of BY4741 gene expression levels under the tested conditions. Diagonal images show density plots of log-transformed fragments per million (FPM) values; (bottom) pairwise scatterplots of gene expression; (top) Pearson correlation coefficients between sample groups.

(C) Volcano plots summarizing the outcome of the differential expression analysis conducted on the RNA-sequencing datasets obtained for each biological replicate under the tested conditions. Highlighted proteins (in red) were deemed differentially expressed; $\log_2$(fold-change) of more than 1 and a false discovery rate (FDR) of $\leq 0.05$.

two genes encoding a mitochondrial aldehyde dehydrogenase (*ALD5*), and a guanosine diphosphate (GDP) dissociation factor (*GDI1*). In addition, ABA treatment increased the levels of three small nucleolar RNAs (snoRNAs; snR5, snR49, and snR80; Figure 3C).

The few differentially expressed genes showed no clear connection to known ABA effects. Notably, *ALD5* (encoding aldehyde dehydrogenase) was upregulated. While trace abscisic aldehyde (AB-ald, an Ald5 substrate and ABA biosynthesis intermediate) in our high-purity ABA ($\geq$98%) could explain this increase, *ALD5* was similarly elevated in methanol-only cultures (fold-change: 1.24; adjusted $p$ value = 0.45; Table S1). Since Ald5 oxidizes formaldehyde (produced from methanol by alcohol dehydrogenases) to formic acid, *ALD5* upregulation likely reflects methanol metabolism rather than ABA exposure.

Consistent with enhanced activity of the mitochondrial aldehyde dehydrogenase, upregulation of the mitochondrial ribosomal RNAs could equally stem from ABA treatment. In plants, ABA is known to alter mitochondrial redox balance, thereby affecting mitochondrial gene expression.[46] Since mitochondrial redox signaling routes are largely conserved among species, it is plausible that such mitochondrial transcriptome changes are, at least partially, echoed in yeast. Ultimately, this could explain why ABA addition increases 21S and 15S rRNA levels,

CellPress
OPEN ACCESS

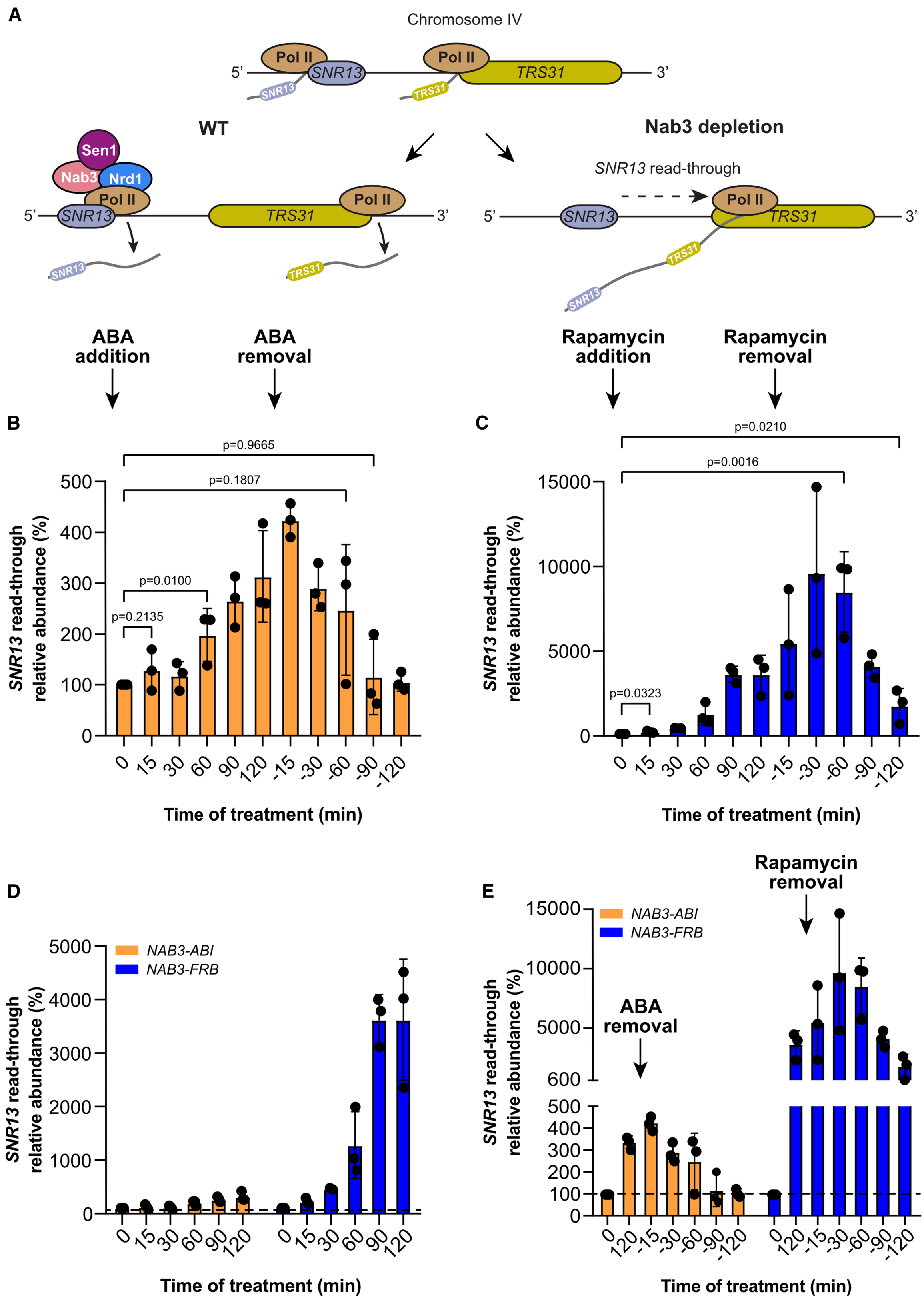


(legend on next page)

which are encoded within the mitochondrial chromosome. Gdi1 regulates the traffic of vesicles by controlling the dissociation of GDP from GTP-binding proteins.[47] Interestingly, in rice, ABA has been shown to induce the synthesis of OsRhoGDI2, a stress-responsive GDI.[48] However, the molecular basis underlying this phenomenon remains uncharacterized.

Thus, ABA concentrations up to 2 mM cause neither growth defects nor major transcriptional changes in budding yeast.

### The ABA-AA system confers fully reversible depletion of Nab3

*NAB3-ABI* required 2000-fold more ligand than did *NAB3-FRB* (500 μM ABA vs. 0.25 μM rapamycin; Figures 2B–2D), indicating that the rapamycin-FKBP12-FRB ternary complex is more stable than the ABA-PYL-ABI complex. This difference reflects the binding affinities underpinning each target-anchor interaction: the ABI-PYL interaction has a $K_D$ of 52 μM in the presence of ABA,[49] whereas the FKBP12-rapamycin-FRB ternary complex binds ~4300-fold more tightly ($K_D$ of ~12 nM).[16] Despite requiring higher ligand concentrations, the weaker PYL-ABA-ABI interaction should enable full reversibility.

To confirm the concentration difference and compare depletion kinetics and reversibility, we performed time-resolved analyses of Nab3 nuclear depletion in both strains. We added ligand to induce depletion, then washed the cells to remove the drug and assess reversibility. To quantify depletion efficiency and reversibility in this time-resolved assay, we exploited the Nrd1-Nab3-Sen1 (NNS) complex requirement for *SNR13* snoRNA termination and 3′ end processing[50] (Figure 4A). When any NNS component is depleted, Pol II transcribes beyond the normal *SNR13* termination site and continues to synthesize species that extend to the downstream *TSR31* gene[50] (Figure 4A). These *SNR13* readthrough products can be quantified by real-time quantitative PCR (RT-qPCR) reaction and have served as a reliable proxy for Nab3, Nrd1, and Sen1 depletion efficiency in previous work.[27,38,51] To determine Nab3 depletion kinetics, we measured 3′ extended *SNR13* levels for two hours following ligand addition (Figures 4B and 4C). To assess reversibility, we washed out rapamycin or ABA and quantified *SNR13* readthrough for an additional two hours (Figures 4B and 4C).

Approximately one hour of ABA treatment was sufficient to detect significant accumulation of 3′ extended *SNR13* species in *NAB3-ABI* cells (Figure 4B). Extended *SNR13* species in *NAB3-FRB* were readily detected after 15 min of rapamycin treatment (Figure 4C). Due to the higher affinity of the FKBP12-rapamycin-FRB ternary complex, rapamycin both depleted Nab3 faster and sequestered a greater fraction of the Nab3 pool compared with ABA (Figure 4D). After two hours of treatment, extended *SNR13* levels in *NAB3-FRB* were 7-fold higher than in *NAB3-ABI* (Figure 4D). However, the rapamycin system showed minimal recovery: *SNR13* processing remained impaired two hours after drug removal (Figures 4C–4E). At this time point, *SNR13* readthrough levels in *NAB3-FRB* were 6-fold higher than pre-treatment levels (Figure 4E). Strikingly, *SNR13* readthrough continued increasing until 90 min after rapamycin removal (Figures 4C–4E). In contrast, 3′ extended *SNR13* in *NAB3-ABI* began decreasing 15 min after ABA withdrawal and returned to pre-treatment levels within 1–1.5 h (Figure 4B).

These assays demonstrate that, despite being slower than the rapamycin-based system (15 min vs. 60 min to achieve significant *SNR13* readthrough), the ABA-AA induced phenotypes that were fully reversed after 90 min. Even though more gradual depletion kinetics may render the ABA-dependent system slightly less suited than its rapamycin-driven counterpart in scenarios requiring nuclear depletion of the target, its reversibility makes the ABA-driven conditional depletion system a powerful tool for studying nuclear protein dynamics. For instance, temporarily retaining a cytoplasmic pool of target protein provides an ideal starting point to study nuclear import kinetics. After inducing partial depletion with ABA, hormone removal allows the target to return to the nucleus, and reimport can be tracked using live-cell fluorescence microscopy (see the following text), such as fluorescence recovery after photobleaching (FRAP),[53] which quantifies the rate and extent of molecular movement by monitoring how quickly fluorescence returns to a bleached region over time. It should also be possible to study transcription dynamics by temporarily titrating transcription factors and/or RNA polymerase subunits from the nucleus to the cytoplasm and following how transcription resumes after their controlled reintroduction. Another advantage of the ABA-AA system in this experimental setup is that, by gradually restoring the native distribution and concentration of the target protein, it avoids saturating the nuclear import machinery and reflects the import-export balance more accurately than methods that fail to reinstate the original steady-state distribution (e.g., the rapamycin-mediated AA).

Although ABA is considerably cheaper per mole than rapamycin, the 500-fold higher concentration required (500 μM vs. 1 μM)

**Figure 4. The ABA-AA system enables fully reversible nuclear depletion of proteins**

(A) Diagrammatic representation of *SNR13* snoRNA termination. *SNR13* (purple) and *TRS31* (green) loci lie on chromosome IV and are transcribed by the RNA polymerase II (Pol II). The Nrd1-Nab3-Sen1 (NNS) complex binds to nascent *SNR13* snoRNAs and Pol II, promoting transcription termination and release of the snoRNA transcript. Assembly of NNS onto their *SNR13* snoRNA targets prevents interference with downstream *TRS31* transcription. Loss of Nab3 results in transcriptional readthrough of Pol II from *SNR13* into *TRS31*.

(B and C) Relative abundances of 3′ extended *SNR13* transcripts were measured using RT-qPCR and normalized to the measurement recorded right before administering 500 μM ABA or 1 μM rapamycin (0 min). The inducer was added at mid-exponential growth phase, and RNA was isolated from samples gathered periodically. After 2 h of treatment, the hormone and the drug were washed away, and RNA was extracted from cells collected at the indicated intervals. Quantification cycles (Cq) were normalized using TidyqPCR,[52] using median Cq values of the *RDN5* and *PGK1* reference genes to calculate the differential Cq (ΔCq) and the relative abundance ($2^{-\Delta Cq}$) for the readthrough transcripts. Each sample was measured in three technical replicates, which were averaged prior to analysis. Shown are means and SDs from $n = 3$ independent biological experiments. $p$ values were calculated using a two-sided ratio paired $t$ test.

(D and E) Relative abundances of 3′ extended *SNR13* transcripts in the *NAB3-ABI* and *NAB3-FRB* strains upon addition (D) and removal (E) of ABA (500 μM) or rapamycin (1 μM), respectively. Each sample was measured in three technical replicates, which were averaged prior to analysis. Shown are means and SDs from $n = 3$ independent biological experiments. $p$ values were calculated using a two-sided paired $t$ test.

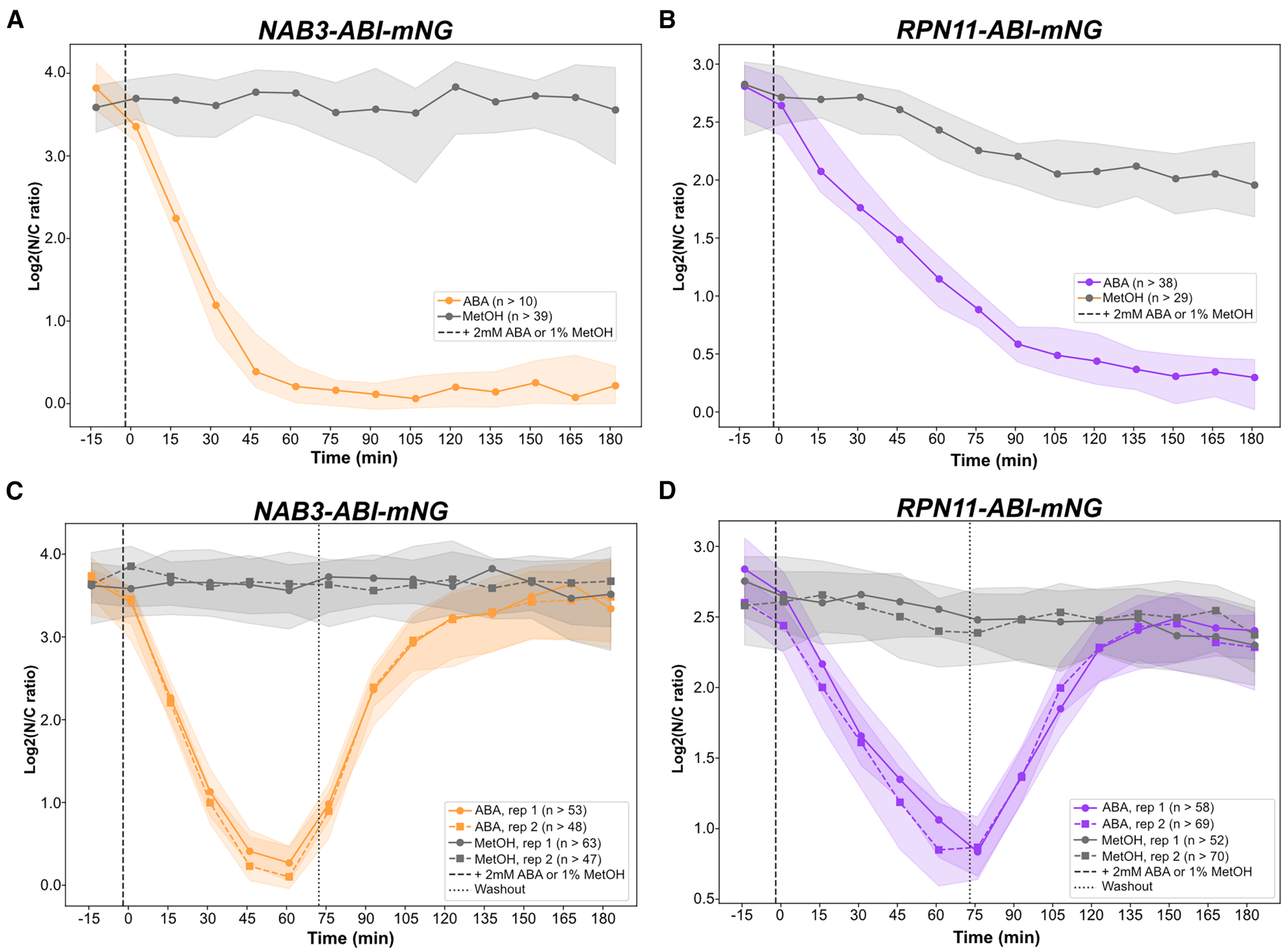


**Figure 5. Nuclear localization dynamics of Nab3-ABI and Rpn11-ABI following ABA treatment and washout**

Nuclear-to-cytosolic concentration ratios for Nab3-ABI-mNeonGreen (A and C) or Rpn11-ABI–mNeonGreen (B and D) are plotted. Points show the median log2 ratio, and error bars show the 25th to 75th percentiles. Cells were treated continuously with ABA (A and B) or subjected to a 73 min pulse followed by ABA washout (C and D). Nuclear and cytosolic protein concentrations were determined as the mean pixel intensity in each compartment, quantified by confocal imaging. Dashed lines denote ABA addition. Dotted lines denote ABA washout. Control cells treated with solvent (methanol) are shown in gray. “n” indicates number of cells in the first frame of the movie. See Figures S2–S5 for representative microscopy images

still results in medium preparation costs that are approximately five times higher per experiment. However, even this cost difference can be eliminated by using 2-cis,4-trans-ABA, a synthetic analog of the hormone that has been used successfully in ABA-mediated CID systems in other organisms.[34] Furthermore, as ABI and PYL tags can be readily introduced by homologous recombination, this system eliminates the need for additional cloning into the HHY168 W303 reference strain or for engineering rapamycin-resistant mutations into the working strain.

## Rapid cytoplasmic sequestration and nuclear restoration of Nab3 using ABA-AA

To confirm that abscisic acid chemically induced dimerization (ABA-CID) effectively depletes proteins from the nucleus, we used live fluorescence microscopy, focusing on the abundant nuclear protein Nab3. mCherry was appended to the C terminus of Pus1 to serve as a nuclear marker, while mNeonGreen (mNG) was fused to the C terminus of Nab3-ABI, yielding the *NAB3-ABI-mNG* strain. Time-lapse imaging was then used to simultaneously monitor and quantify the localization of Nab3 and Pus1 in the presence of 2 mM ABA or 1% solvent (MetOH). Figure 5 shows the quantification of the results. Representative microscopy images are provided in Figures S2 and S4. Approximately 90% of the nuclear Nab3 fluorescence signal was lost within one hour of ABA treatment. After 75 min, the detectable signal was almost entirely cytoplasmic (Figure 5A; Figure S2A), confirming near-complete cytoplasmic sequestration of Nab3-ABI. MetOH treatment did not impact Nab3 nuclear localization (Figure 5A; Figure S2B). These data, therefore, demonstrate that the observed growth and *SNR13* processing defects observed in the *NAB3-ABI* strain treated with ABA are a direct result of cytoplasmic sequestration of Nab3.

Next, to assess the reversibility of ABA-induced depletion at the single-cell level, we continued imaging after washing ABA out of the medium. After exposing cells to 2 mM ABA for 73 min, we monitored nuclear signal restoration by time-lapse microscopy at 15 min intervals (Figure 5C; Figures S3A and S3B). This revealed that nuclear localization of the Nab3 reporter was fully restored in

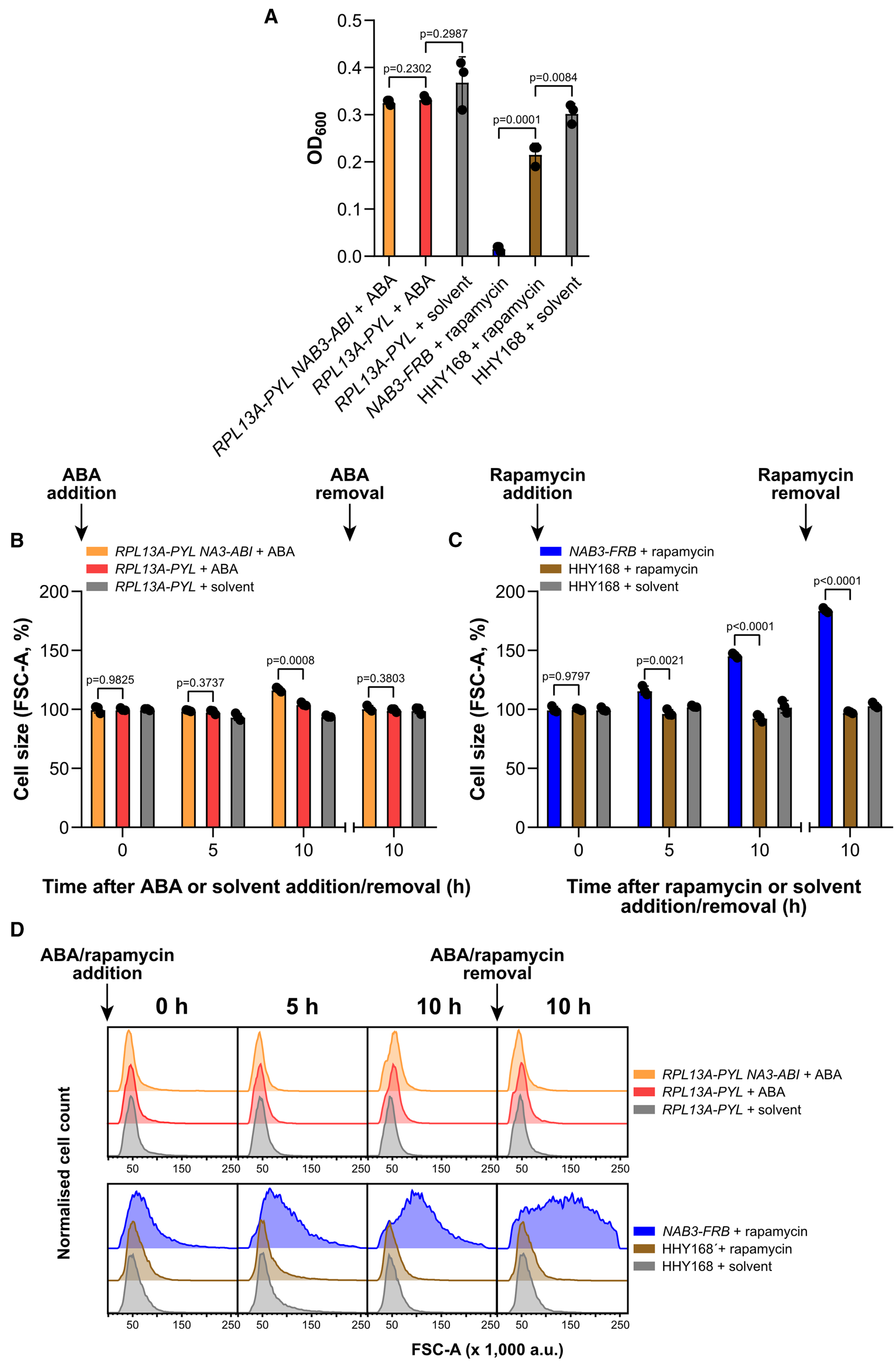


(legend on next page)

approximately an hour, roughly the same time it took to fully deplete Nab3 from the nucleus in the presence of ABA.

### Anomalies caused by ABA-induced Nab3 depletion are fully reversible

Our microscopy data provide direct visual evidence that the ABA-mediated AA system effectively titrates an abundant nuclear protein to the cytoplasm and that, upon removal of the hormone, the proteins rapidly return to the nucleus. Yet, molecular re-localization and functional recovery need not be synchronous. Cells may take considerably longer to re-establish normal physiology once a protein has returned to its compartment or may fail to do so altogether. For researchers using ABA-AA to study nuclear proteins with defined physiological roles, these are important practical questions: how long after hormone removal does it take for cells to resume normal function, and is recovery complete?

To address this, we examined a well-characterized physiological consequence of Nab3 depletion as a model readout. We previously showed that prolonged cytoplasmic sequestration of Nab3, using rapamycin-mediated AA, causes a significant increase in cell size when cells are grown in medium containing a low concentration of a non-fermentable carbon source.[25] This offered a convenient physiological readout, and the reversibility of ABA-AA made it possible to ask directly whether the cell-size phenotype itself is reversible upon restoration of normal Nab3 localization. To test this, we (i) monitored the forward scatter area (FSC-A) as a semi-quantitative indicator of cell size in *NAB3-ABI* and *NAB3-FRB* cultures for 10 h during ABA or rapamycin treatment, respectively, and (ii) re-analyzed both cultures 10 h after compound removal (the "recovery phase"). We also evaluated forward scatter in two control strains: *RPL13A-PYL* (anchor only) and HHY168 (rapamycin-resistant parental strain) treated only with the corresponding solvents (i.e., 1% methanol and 1% ethanol, respectively). After 10 h of recovery, *NAB3-ABI* and control strain *RPL13A-PYL* reached exponential phase, whereas *NAB3-FRB* remained growth-arrested (Figure 6A), confirming rapamycin-based AA irreversibility within the tested time frame. ABA-treated *RPL13A-PYL* grew identically to solvent controls, while rapamycin-treated HHY168 showed mild growth inhibition compared with ethanol-only controls, demonstrating rapamycin toxicity independent of target depletion. Thus, unlike rapamycin, ABA-AA is not toxic.

Flow cytometry showed that *NAB3-FRB* forward scatter increased by 16% and 45% at 5 and 10 h of rapamycin treatment, respectively. Unexpectedly, cells continued enlarging during recovery, reaching 80% above their initial measurement (Figures 6B and 6C), comparable to cell size increases seen with conditional depletion of other NNS components.[25,29,54,55] Since HHY168 showed no dimensional change throughout the experiment (Figures 6B–6D), we conclude that rapamycin itself does not cause cell enlargement in the rapamycin-resistant HHY168 background. Thus, the persistent increase in FSC-A in *NAB3-FRB* confirms that the drug remains bound to the complex for a significant time after washing away the drug and the Nab3 rapamycin-based AA is not fully reversible.

*NAB3-ABI* forward scatter profile remained unchanged for the first 5 h of ABA treatment but increased by ∼16% at 10 h (Figures 6B and 6C). Importantly, *NAB3-ABI* returned to normal dimensions within 10 h of ABA removal (Figures 6B–6D), confirming full reversibility of ABA-AA. These results demonstrate that ABA-AA can be used not only to effectively deplete nuclear proteins of interest but also to assess whether phenotypes arising from their loss are fully reversible, a question that is difficult to address cleanly with other approaches.

### The ABA-AA system is applicable to abundant nuclear proteins

The effectiveness of any CID technique depends on the abundance of the target protein relative to that of the anchor. Rpl13A is present at around 50,000 molecules per cell, making it roughly 9 times more abundant than Nab3 (Figure 7A; Table 1). Although Nab3 is among the most abundant proteins (80th percentile; Figure 7A; Table S2), we tested whether ABA-AA could deplete even more abundant proteins.

We generated an *RPL13A-PYL* strain with ABI fused to the essential proteasome component Rpn11, which is present at ∼16,000 copies per cell (95th percentile; Figure 7A; Table 1; Table S2). We then performed growth assays on agar plates and in liquid culture containing 500 μM or 2 mM ABA (Figures 7B and 7C), with *NAB3-ABI* as a positive control and BY4741 as a negative control. *RPN11-ABI* grew normally without ABA, indicating that the tag does not impair function. Consistent with its greater molecular count, Rpn11 appeared to be less efficiently depleted from the nucleus than Nab3. Notably, increasing the ABA concentration to 2 mM exacerbated the growth defect of the *RPN11-ABI* strain (Figures 7B and 7C). In agreement with previous technical recommendations,[15,20,21] this observation reinforced the importance of optimizing the inducer concentration to achieve target protein abundance. Given that the aqueous solubility of ABA (0.3 mg/mL) is superior to that of rapamycin (2.6 μg/mL), the former can be added to the medium in concentrations far exceeding those reported here, with no risk of precipitation even at 2 mM. Since both tested concentrations of ABA induced substantial *RPN11-ABI* growth defects (Figures 7B and 7C), ABA-AA can deplete even highly abundant proteins, providing a valuable alternative to rapamycin-based AA.

**Figure 6. Growth defects and dimensional anomalies arising from ABA-induced nuclear depletion are fully reversible**

(A) Recorded optical densities of *NAB3-ABI* and *NAB3-FRB* and parental strain cultures 10 h after removing ABA (500 μM) or rapamycin (1 μM) from the medium. Lines and shading represent the means and SDs of three technical replicates.

(B and C) Weighted means and SDs of cell size (FSC-A values in flow cytometry) for $n$ = 3 independent biological repeats of NAB3-ABI, RPL13A-PYL, NAB3-FRB, and HHY168, measured before treatment (0 h); after 5 and 10 h of ABA (500 μM), rapamycin (1 μM), or solvent addition; and 10 h after compound removal (recovery). Values were normalized to the pre-treatment measurement for each strain. $p$ values were calculated using a two-sided unpaired Student's $t$ test.

(D) Flow cytometry traces of the FSC-A distribution of *NAB3-ABI* and *NAB3-FRB* and their parental strains treated with the indicated dimerization agent or its solvent (methanol or ethanol) alone. Representative of $n$ = 3 independent biological repeats.

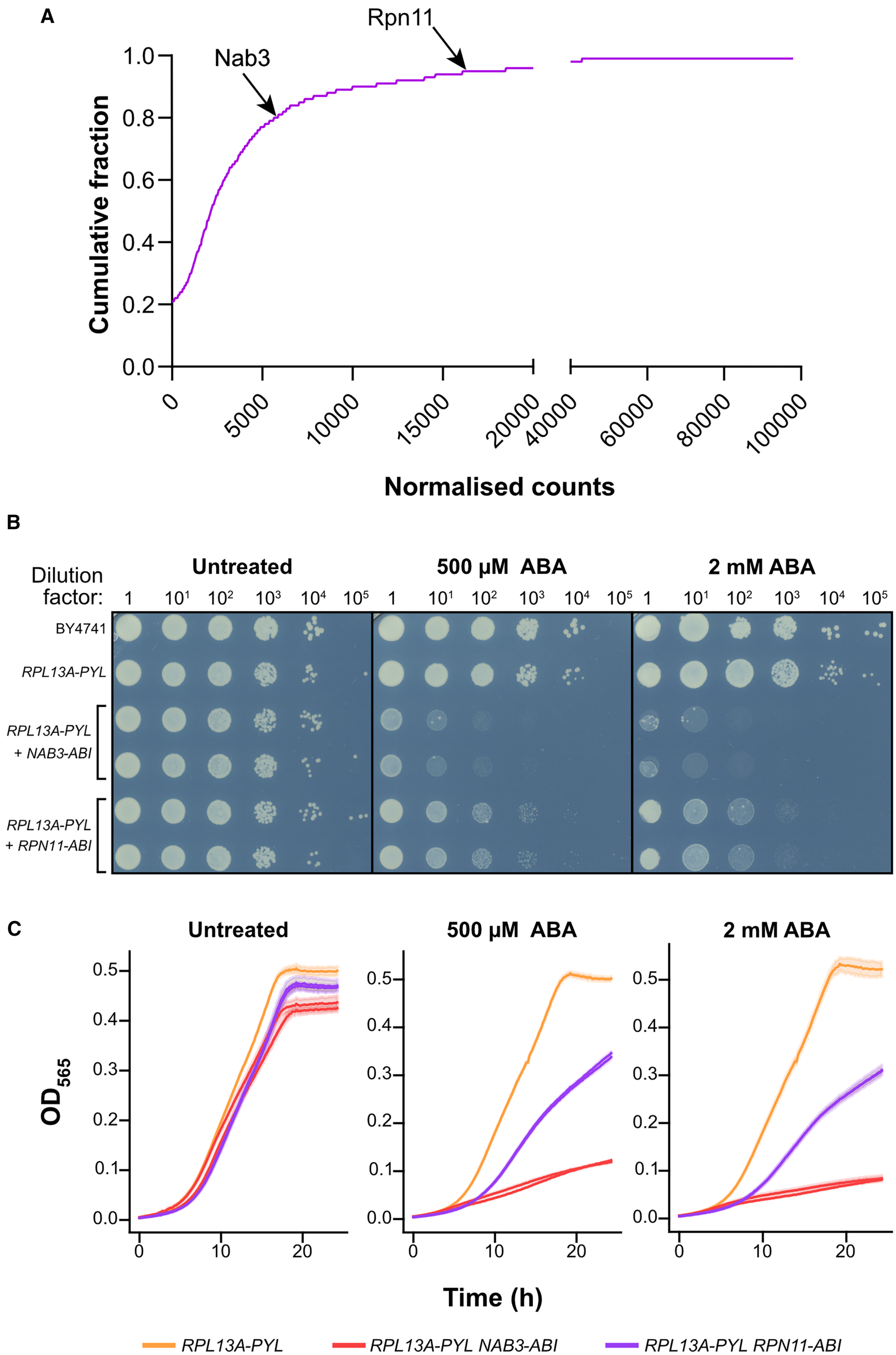


*(legend on next page)*

To verify that the ABA-AA system achieves complete cytoplasmic sequestration and full rapid restoration of highly abundant nuclear proteins, we applied our time-resolved live-cell microscopy approach described earlier to a *RPN11-ABI-mNG* (*RPL13A-PYL-3xHA RPN11-ABI-3xFLAG-mNeonGreen PUS1-mCherry)* strain. As with Nab3-ABI, the green signal emitted by Rpn11-ABI became predominantly cytoplasmic within 76 min of supplementing the medium with 2 mM ABA and relocated to the nucleus within an hour of hormone removal (Figures 5B and 5D; Figures S4A and S4B; Figures S5A and S5B). Together, these results demonstrate that the ABA-AA system achieves complete nuclear depletion of even highly abundant target proteins and permits their full and rapid restoration following hormone withdrawal.

In summary, we have shown that ABA-AA efficiently depletes nuclear proteins in a fully reversible manner, even when the target is highly abundant. The rapidity of both depletion and restoration makes the system particularly well suited for studying dynamic nuclear processes and for dissecting the kinetic relationship between protein localization and cellular phenotypes, including whether those phenotypes are themselves reversible. Unlike rapamycin, ABA is non-toxic to wild-type yeast, does not dramatically alter gene expression, and is suitable for diverse genetic backgrounds. Requiring only two fusion proteins, ABA-AA provides a non-toxic and broadly applicable alternative for conditional and reversible protein depletion in budding yeast and other eukaryotes.

### Limitations of the study

The ABA-AA method has certain limitations that should be considered. Although ABA is largely innocuous to *S. cerevisiae* and does not cause major transcriptional changes at the concentrations used, it is not completely neutral; we observed modest effects on the expression of mitochondrial rRNAs and a small set of nuclear genes, indicating that chronic ABA exposure may subtly influence mitochondrial function or redox homeostasis. The ABA-PYL-ABI interaction is considerably weaker than the FKBP12-rapamycin-FRB complex, necessitating relatively high micromolar to millimolar hormone concentrations and resulting in slower depletion of Nab3 compared with the rapamycin-based AA. This slower onset may limit the applicability of ABA-AA in experimental setups that require very rapid protein removal from the nucleus, but it also underlies the full reversibility of the system, which is difficult to achieve with rapamycin-mediated depletion. Although we showed that ABA-AA can be effectively applied to very highly abundant nuclear proteins such as Rpn11, efficient depletion in such cases required higher ABA concentrations and/or longer incubation times, and optimal conditions are likely to be target- and context-dependent.

The original rapamycin-dependent AA system demonstrated that yeast histone (H2B) and membrane-bound proteins (Pma1) can also serve as effective anchors.[14] We have not yet assessed whether such alternative anchors are compatible with ABA-AA. Since all functional interference observed here was achieved using a ribosomal anchor, we cannot completely rule out the possibility that some of the observed phenotypic effects reflect steric interference arising from tethering the target protein to translating ribosomes rather than its removal from the nucleus per se.

Finally, it is important to note that the ABI domain is relatively large and, therefore, C-terminal tagging can partially compromise protein function.

## RESOURCE AVAILABILITY

### Lead contact

Requests for further information and resources should be directed to and will be fulfilled by the lead contact, Sander Granneman (sander.granneman@ed.ac.uk).

### Materials availability

All yeast strains and plasmids are available from the lead contact, Sander Granneman (sander.granneman@ed.ac.uk).

### Data and code availability

- The sequencing data are available from the Gene Expression Omnibus (GEO accession GSE149746).
- The pyCRAC package and the pipeline used to analyze the RNA sequencing data are available from https://git.ecdf.ed.ac.uk/sgrannem/pycrac, from PyPi https://pypi.org/project/pyCRAC/, and from https://git.ecdf.ed.ac.uk/sgrannem/crac_pipelines.
- Any additional information required to re-analyze the data reported in this paper is available from the lead contact upon request.

## ACKNOWLEDGMENTS

We are grateful to Isabella Maudlin, Jean Beggs, Manu Shakla, Priya Amin, and Kevin Hardwick for providing reagents and plasmids and for fruitful discussions. We thank the members of the Swain and Granneman laboratories for critically reading the manuscript. Flow cytometry data were generated within the flow cytometry and cell sorting facility in the Ashworth Building at the University of Edinburgh. The facility was supported by funding from the Wellcome Trust and the University of Edinburgh. This work was supported by funding for the Wellcome Discovery Research Platform for Hidden Cell Biology (226791), and we gratefully acknowledge support from the Light Microscopy core. This work was supported by a Medical Research Council Programme grant (MR/Y013131/1) to S.G., a SynBio-Med Wellcome Trust institutional fund to S.G., a Wellcome Trust Career Development Award (225903/Z/22/Z) to M.P.S., a Wellcome Trust PhD training fellowship (224084/Z/21/Z) to S.E.-S., an Erasmus+ traineeship to T.W., and a Darwin Trust of Edinburgh PhD Award to S.S.

**Figure 7. ABA-dependent AA can deplete even the most highly abundant nuclear proteins**

(A) Cumulative distribution of molecules per cell for the *Saccharomyces cerevisiae*[43,56] nuclear proteome (Table S2). Indicated are the abundances of Nab3 and Rpn11.

(B) Spot test analyses testing the efficiency of the ABA-AA system in selectively relocalizing Rpn11. *NAB3-ABI* served as a positive control for detecting defects in the presence of ABA. The BY4741 reference and the parental *RPL13A-PYL* strain served as negative controls. Serial dilutions of cells were spotted onto plates containing minimal medium supplemented with glucose and the indicated dose of ABA or rapamycin.

(C) Time-resolved optical density measurements depicting the mean (line) and the standard deviation (shading) of three technical repeats. Two independent clones were examined for the *RPN11-ABI* and *NAB3-ABI* strains.

## AUTHOR CONTRIBUTIONS

Conceptualization, methodology, supervision, and funding acquisition, S.G. and M.P.S.; investigation, T.W., S.E.-S., H.G., S.S., and I.F.; visualization, S.E.-S., T.W., H.G., S.S., and S.G.; writing – original draft, S.E.-S. and S.G.; writing – review and editing, all authors.

## DECLARATION OF INTERESTS

The authors have no interests to declare.

## DECLARATION OF GENERATIVE AI AND AI-ASSISTED TECHNOLOGIES IN THE WRITING PROCESS

During the preparation of this work, the authors used Claude Sonnet to correct spelling and grammar mistakes and improve the clarity of the text. After using this tool, the authors reviewed and edited the content as needed and take full responsibility for the content of the published article.

## STAR★METHODS

Detailed methods are provided in the online version of this paper and include the following:

- KEY RESOURCES TABLE
- EXPERIMENTAL MODEL AND STUDY PARTICIPANT DETAILS
  - Strains and growth conditions
- METHOD DETAILS
  - Spot-test growth assays
  - Microplate reader growth assays
  - RNA extraction
  - RNA sequencing
  - RNA-sequencing analysis
  - Real-time quantitative reverse transcription PCR
  - Cell size measurements using flow cytometry
  - Flow cytometry analyses
  - Microscopy-based assessment of ABA anchor-away nuclear depletion and recovery
- QUANTIFICATION AND STATISTICAL ANALYSIS

## SUPPLEMENTAL INFORMATION

Supplemental information can be found online at https://doi.org/10.1016/j.chembiol.2026.06.003.

Received: November 22, 2025
Revised: May 13, 2026
Accepted: June 10, 2026
Published: July 8, 2026

## REFERENCES

1. Johnston, M., and Davis, R.W. (1984). Sequences that regulate the divergent GAL1-GAL10 promoter in Saccharomyces cerevisiae. Mol. Cell Biol. *4*, 1440–1448. https://doi.org/10.1128/mcb.4.8.1440.
2. Mountain, H.A., Byström, A.S., Larsen, J.T., and Korch, C. (1991). Four major transcriptional responses in the methionine/threonine biosynthetic pathway of Saccharomyces cerevisiae. Yeast *7*, 781–803. https://doi.org/10.1002/yea.320070804.
3. Gnanasundram, S.V., and Koš, M. (2015). Fast protein-depletion system utilizing tetracycline repressible promoter and N-end rule in yeast. Mol. Biol. Cell *26*, 762–768. https://doi.org/10.1091/mbc.E14-07-1186.
4. Bellí, G., Garí, E., Piedrafita, L., Aldea, M., and Herrero, E. (1998). An activator/repressor dual system allows tight tetracycline-regulated gene expression in budding yeast. Nucleic Acids Res. *26*, 942–947. https://doi.org/10.1093/nar/26.4.942.
5. Garí, E., Piedrafita, L., Aldea, M., and Herrero, E. (1997). A set of vectors with a tetracycline-regulatable promoter system for modulated gene expression in Saccharomyces cerevisiae. Yeast *13*, 837–848. https://doi.org/10.1002/(SICI)1097-0061(199707)13:9<837::AID-YEA145>3.0.CO;2-T.
6. Kötter, P., Weigand, J.E., Meyer, B., Entian, K.D., and Suess, B. (2009). A fast and efficient translational control system for conditional expression of yeast genes. Nucleic Acids Res. *37*, e120. https://doi.org/10.1093/nar/gkp578.
7. Suess, B., Entian, K.D., Kötter, P., and Weigand, J.E. (2012). Aptamer-regulated expression of essential genes in yeast. Methods Mol. Biol. *824*, 381–391. https://doi.org/10.1007/978-1-61779-433-9_20.
8. Stelter, P., and Hurt, E. (2014). A pulse-chase epitope labeling to study cellular dynamics of newly synthesized proteins: a novel strategy to characterize NPC biogenesis and ribosome maturation/export. Methods Cell Biol. *122*, 147–163. https://doi.org/10.1016/B978-0-12-417160-2.00007-2.
9. Nishimura, K., Fukagawa, T., Takisawa, H., Kakimoto, T., and Kanemaki, M. (2009). An auxin-based degron system for the rapid depletion of proteins. Nat. Methods *6*, 917–922. https://doi.org/10.1038/nmeth.1401.
10. Li, S., Prasanna, X., Salo, V.T., Vattulainen, I., and Ikonen, E. (2019). An efficient auxin-inducible degron system with low basal degradation in human cells. Nat. Methods *16*, 866–869. https://doi.org/10.1038/s41592-019-0512-x.
11. Ding, L., Laor, D., Weisman, R., and Forsburg, S.L. (2014). Rapid regulation of nuclear proteins by rapamycin-induced translocation in fission yeast. Yeast *31*, 253–264. https://doi.org/10.1002/yea.3014.
12. Kanke, M., Nishimura, K., Kanemaki, M., Kakimoto, T., Takahashi, T.S., Nakagawa, T., and Masukata, H. (2011). Auxin-inducible protein depletion system in fission yeast. BMC Cell Biol. *12*, 8. https://doi.org/10.1186/1471-2121-12-8.
13. Sathyan, K.M., McKenna, B.D., Anderson, W.D., Duarte, F.M., Core, L., and Guertin, M.J. (2019). An improved auxin-inducible degron system preserves native protein levels and enables rapid and specific protein depletion. Genes Dev. *33*, 1441–1455. https://doi.org/10.1101/gad.328237.119.
14. Haruki, H., Nishikawa, J., and Laemmli, U.K. (2008). The Anchor-Away Technique: Rapid, Conditional Establishment of Yeast Mutant Phenotypes. Mol. Cell *31*, 925–932. https://doi.org/10.1016/J.MOLCEL.2008.07.020.
15. Mendoza-Ochoa, G.I., Barrass, J.D., Terlouw, B.R., Maudlin, I.E., de Lucas, S., Sani, E., Aslanzadeh, V., Reid, J.A.E., and Beggs, J.D. (2019). A fast and tuneable auxin-inducible degron for depletion of target proteins in budding yeast. Yeast *36*, 75–81. https://doi.org/10.1002/yea.3362.
16. Banaszynski, L.A., Liu, C.W., and Wandless, T.J. (2005). Characterization of the FKBP-rapamycin-FRB ternary complex. J. Am. Chem. Soc. *127*, 4715–4721. https://doi.org/10.1021/ja043277y.
17. Ban, N., Beckmann, R., Cate, J.H., Dinman, J.D., Dragon, F., Ellis, S.R., Lafontaine, D.L., Lindahl, L., Liljas, A., Lipton, J.M., et al. (2014). A new system for naming ribosomal proteins. Curr. Opin. Struct. Biol. *24*, 165–169. https://doi.org/10.1016/j.sbi.2014.01.002.
18. Papagiannakis, A., De Jonge, J.J., Zhang, Z., and Heinemann, M. (2017). Quantitative characterization of the auxin-inducible degron: a guide for dynamic protein depletion in single yeast cells. Sci. Rep. *7*, 4704. https://doi.org/10.1038/s41598-017-04791-6.
19. Natsume, T., Kiyomitsu, T., Saga, Y., and Kanemaki, M.T. (2016). Rapid Protein Depletion in Human Cells by Auxin-Inducible Degron Tagging with Short Homology Donors. Cell Rep. *15*, 210–218. https://doi.org/10.1016/j.celrep.2016.03.001.
20. Yesbolatova, A., Saito, Y., Kitamoto, N., Makino-Itou, H., Ajima, R., Nakano, R., Nakaoka, H., Fukui, K., Gamo, K., Tominari, Y., et al. (2020). The auxin-inducible degron 2 technology provides sharp degradation control in yeast, mammalian cells, and mice. Nat. Commun. *11*, 5701–5713. https://doi.org/10.1038/S41467-020-19532-Z.
21. Barrass, J.D., Mendoza-Ochoa, G.I., Maudlin, I.E., Sani, E., and Beggs, J.D. (2019). Tuning degradation to achieve specific and efficient protein depletion. J. Vis. Exp. *149*, e59874. https://doi.org/10.3791/59874.

22. Arevalo-Rodriguez, M., Wu, X., Hanes, S.D., and Heitman, J. (2004). Prolyl isomerases in yeast. Front. Biosci. *9*, 2420–2446. https://doi.org/10.2741/1405.

23. Heitman, J., Movva, N.R., and Hall, M.N. (1991). Targets for cell cycle arrest by the immunosuppressant rapamycin in yeast. Science *253*, 905–909. https://doi.org/10.1126/SCIENCE.1715094.

24. Millson, S.H., and Piper, P.W. (2014). Insights from yeast into whether the inhibition of heat shock transcription factor (Hsf1) by rapamycin can prevent the Hsf1 activation that results from treatment with an Hsp90 inhibitor. Oncotarget *5*, 5054–5064. https://doi.org/10.18632/oncotarget.2077.

25. Esteban-Serna, S., Widén, T., Gwynne, M., Farquhar, I., Duchen, M.R., Swain, P.S., and Granneman, S. (2025). A transcription termination mechanism for maintaining homogeneous protein expression. Nucleic Acids Res. *53*, gkaf1199. https://doi.org/10.1093/NAR/GKAF1199.

26. van Nues, R., Schweikert, G., De Leau, E., Selega, A., Langford, A., Franklin, R., Iosub, I., Wadsworth, P., Sanguinetti, G., and Granneman, S. (2017). Kinetic CRAC uncovers a role for Nab3 in determining gene expression profiles during stress. Nat. Commun. *8*, 12. https://doi.org/10.1038/s41467-017-00025-5.

27. Bresson, S., Tuck, A., Staneva, D., and Tollervey, D. (2017). Nuclear RNA Decay Pathways Aid Rapid Remodeling of Gene Expression in Yeast. Mol. Cell *65*, 787–800.e5. https://doi.org/10.1016/j.molcel.2017.01.005.

28. Thiebaut, M., Colin, J., Neil, H., Jacquier, A., Séraphin, B., Lacroute, F., and Libri, D. (2008). Futile cycle of transcription initiation and termination modulates the response to nucleotide shortage in S. cerevisiae. Mol. Cell *31*, 671–682. https://doi.org/10.1016/j.molcel.2008.08.010.

29. Darby, M.M., Serebreni, L., Pan, X., Boeke, J.D., and Corden, J.L. (2012). The Saccharomyces cerevisiae Nrd1-Nab3 Transcription Termination Pathway Acts in Opposition to Ras Signaling and Mediates Response to Nutrient Depletion. Mol. Cell Biol. *32*, 1762–1775. https://doi.org/10.1128/MCB.00050-12.

30. Merran, J., and Corden, J.L. (2017). Yeast RNA-Binding Protein Nab3 Regulates Genes Involved in Nitrogen Metabolism. Mol. Cell Biol. *37*, e00154.17. https://doi.org/10.1128/MCB.00154-17.

31. Liang, F.S., Ho, W.Q., and Crabtree, G.R. (2011). Engineering the ABA Plant Stress Pathway for Regulation of Induced Proximity. Sci. Signal. *4*, rs2. https://doi.org/10.1126/SCISIGNAL.2001449.

32. Cutler, S.R., Rodriguez, P.L., Finkelstein, R.R., and Abrams, S.R. (2010). Abscisic Acid: Emergence of a Core Signaling Network. Annu. Rev. Plant Biol. *61*, 651–679. https://doi.org/10.1146/annurev-arplant-042809-112122.

33. Amin, P., Soper Ní Chafraidh, S., Leontiou, I., and Hardwick, K.G. (2019). Regulated reconstitution of spindle checkpoint arrest and silencing through chemically induced dimerisation in vivo. J. Cell Sci. *132*, jcs219766. https://doi.org/10.1242/jcs.219766.

34. Suresh, A.P., Cheng, H., Davies, T., Clark, I., Spanos, C., and Hardwick, K.G. (2025). Cryptococcus neoformans employs both PP1 and PP2A phosphatases to silence the spindle checkpoint. Preprint at bioRxiv. https://doi.org/10.1101/2025.11.04.686612.

35. Tudek, A., Schmid, M., Makaras, M., Barrass, J.D., Beggs, J.D., and Jensen, T.H. (2018). A Nuclear Export Block Triggers the Decay of Newly Synthesized Polyadenylated RNA. Cell Rep. *24*, 2457–2467.e7. https://doi.org/10.1016/j.celrep.2018.07.103.

36. Sun, M., Schwalb, B., Pirkl, N., Maier, K., Schenk, A., Failmezger, H., Tresch, A., and Cramer, P. (2013). Global analysis of eukaryotic mRNA degradation reveals Xrn1-dependent buffering of transcript levels. Mol. Cell *52*, 52–62. https://doi.org/10.1016/j.molcel.2013.09.010.

37. Schulz, D., Schwalb, B., Kiesel, A., Baejen, C., Torkler, P., Gagneur, J., Soeding, J., and Cramer, P. (2013). Transcriptome surveillance by selective termination of noncoding RNA synthesis. Cell *155*, 1075–1087. https://doi.org/10.1016/j.cell.2013.10.024.

38. Schaughency, P., Merran, J., and Corden, J.L. (2014). Genome-Wide Mapping of Yeast RNA Polymerase II Termination. PLoS Genet. *10*, e1004632. https://doi.org/10.1371/journal.pgen.1004632.

39. Rohde, J., Heitman, J., and Cardenas, M.E. (2001). The TOR Kinases Link Nutrient Sensing to Cell Growth. J. Biol. Chem. *276*, 9583–9586. https://doi.org/10.1074/jbc.R000034200.

40. Cardenas, M.E., Cutler, N.S., Lorenz, M.C., Di Como, C.J., and Heitman, J. (1999). The TOR signaling cascade regulates gene expression in response to nutrients. Genes Dev. *13*, 3271–3279. https://doi.org/10.1101/gad.13.24.3271.

41. Carroll, K.L., Pradhan, D.A., Granek, J.A., Clarke, N.D., and Corden, J.L. (2004). Identification of cis Elements Directing Termination of Yeast Nonpolyadenylated snoRNA Transcripts. Mol. Cell Biol. *24*, 6241–6252. https://doi.org/10.1128/MCB.24.14.6241-6252.2004.

42. Petrezselyova, S., Zahradka, J., and Sychrova, H. (2010). Saccharomyces cerevisiae BY4741 and W303-1A laboratory strains differ in salt tolerance. Fungal Biol. *114*, 144–150. https://doi.org/10.1016/j.funbio.2009.11.002.

43. Ho, B., Baryshnikova, A., and Brown, G.W. (2018). Unification of Protein Abundance Datasets Yields a Quantitative Saccharomyces cerevisiae Proteome. Cell Syst. *6*, 192–205.e3. https://doi.org/10.1016/j.cels.2017.12.004.

44. Webb, S., Hector, R.D., Kudla, G., and Granneman, S. (2014). PAR-CLIP data indicate that Nrd1-Nab3-dependent transcription termination regulates expression of hundreds of protein coding genes in yeast. Genome Biol. *15*, R8. https://doi.org/10.1186/gb-2014-15-1-r8.

45. Love, M.I., Huber, W., and Anders, S. (2014). Moderated estimation of fold change and dispersion for RNA-seq data with DESeq2. Genome Biol. *15*, 550. https://doi.org/10.1186/s13059-014-0550-8.

46. Li, S., Liu, S., Zhang, Q., Cui, M., Zhao, M., Li, N., Wang, S., Wu, R., Zhang, L., Cao, Y., and Wang, L. (2022). The interaction of ABA and ROS in plant growth and stress resistances. Front. Plant Sci. *13*, 1050132. https://doi.org/10.3389/FPLS.2022.1050132.

47. Garrett, M.D., Zahner, J.E., Cheney, C.M., and Novick, P.J. (1994). GDI1 encodes a GDP dissociation inhibitor that plays an essential role in the yeast secretory pathway. EMBO J. *13*, 1718–1728. https://doi.org/10.1002/j.1460-2075.1994.tb06436.x.

48. Huang, J.J., Zhang, J., Hao, Y.F., Yan, X.T., Shi, J., Wang, G.H., Du, J.Y., Ge, H.W., Wang, H.H., and Liang, W.H. (2016). Distinct expression patterns of the GDP dissociation inhibitor protein gene (OsRhoGDI2) from Oryza sativa during development and abiotic stresses. Biologia *71*, 1230–1239. https://doi.org/10.1515/biolog-2016-0146.

49. Miyazono, K.I., Miyakawa, T., Sawano, Y., Kubota, K., Kang, H.J., Asano, A., Miyauchi, Y., Takahashi, M., Zhi, Y., Fujita, Y., et al. (2009). Structural basis of abscisic acid signalling. Nature *462*, 609–614. https://doi.org/10.1038/nature08583.

50. Steinmetz, E.J., Conrad, N.K., Brow, D.A., and Corden, J.L. (2001). RNA-binding protein Nrd1 directs poly(A)-independent 3′-end formation of RNA polymerase II transcripts. Nature *413*, 327–331. https://doi.org/10.1038/35095090.

51. Terzi, N., Churchman, L.S., Vasiljeva, L., Weissman, J., and Buratowski, S. (2011). H3K4 Trimethylation by Set1 Promotes Efficient Termination by the Nrd1-Nab3-Sen1 Pathway. Mol. Cell Biol. *31*, 3569–3583. https://doi.org/10.1128/mcb.05590-11.

52. Wallace, E.W.J., and Haynes, S.J. (2022). tidyqpcr: Quantitative PCR analysis in the tidyverse. J. Open Source Softw. *7*, 4507. https://doi.org/10.21105/joss.04507.

53. Axelrod, D., Koppel, D.E., Schlessinger, J., Elson, E., and Webb, W.W. (1976). Mobility measurement by analysis of fluorescence photobleaching recovery kinetics. Biophys. J. *16*, 1055–1069. https://doi.org/10.1016/S0006-3495(76)85755-4.

54. Ajazi, A., Choudhary, R., Tronci, L., Bachi, A., and Bruhn, C. (2022). CTP sensing and Mec1 ATR-Rad53 CHK1/CHK2 mediate a two-layered response to inhibition of glutamine metabolism. PLoS Genet. *18*, e1010101. https://doi.org/10.1371/journal.pgen.1010101.

55. Aiello, U., Challal, D., Wentzinger, G., Lengronne, A., Appanah, R., Pasero, P., Palancade, B., and Libri, D. (2022). Sen1 is a key regulator of

transcription-driven conflicts. Mol. Cell *82*, 2952–2966.e6. https://doi.org/10.1016/J.MOLCEL.2022.06.021.

56. Huh, W.K., Falvo, J.V., Gerke, L.C., Carroll, A.S., Howson, R.W., Weissman, J.S., and O'Shea, E.K. (2003). Global analysis of protein localization in budding yeast. Nature *425*, 686–691. https://doi.org/10.1038/NATURE02026.

57. Baker Brachmann, C., Davies, A., Cost, G.J., Caputo, E., Li, J., Hieter, P., and Boeke, J.D. (1998). Designer deletion strains derived from Saccharomyces cerevisiae S288C: A useful set of strains and plasmids for PCR-mediated gene disruption and other applications. Yeast *14*, 115–132. https://doi.org/10.1002/(SICI)1097-0061(19980130)14:2<115::AID-YEA204>3.0.CO;2-2.

58. Shishkin, A.A., Giannoukos, G., Kucukural, A., Ciulla, D., Busby, M., Surka, C., Chen, J., Bhattacharyya, R.P., Rudy, R.F., Patel, M.M., et al. (2015). Simultaneous generation of many RNA-seq libraries in a single reaction. Nat. Methods *12*, 323–325. https://doi.org/10.1038/nmeth.3313.

59. Dodt, M., Roehr, J., Ahmed, R., and Dieterich, C. (2012). FLEXBAR — Flexible Barcode and Adapter Processing for Next-Generation Sequencing Platforms. Biology *1*, 895–905. https://doi.org/10.3390/biology1030895.

60. Wickham, H. (2016). ggplot2: Elegant Graphics for Data Analysis, Second ed. (Springer).

61. Schneider, C.A., Rasband, W.S., and Eliceiri, K.W. (2012). NIH Image to ImageJ: 25 years of image analysis. Nat. Methods *9*, 671–675. https://doi.org/10.1038/nmeth.2089.

62. Hunter, J.D. (2007). Matplotlib: A 2D graphics environment. Comput. Sci. Eng. *9*, 90–95. https://doi.org/10.1109/MCSE.2007.55.

63. Montaño-Gutierrez, L.F., Moreno, N.M., Farquhar, I.L., Huo, Y., Bandiera, L., and Swain, P.S. (2022). Analysing and meta-analysing time-series data of microbial growth and gene expression from plate readers. PLoS Comput. Biol. *18*, e1010138. https://doi.org/10.1371/JOURNAL.PCBI.1010138.

64. Waskom, M. (2021). seaborn: statistical data visualization. J. Open Source Softw. *6*, 3021. https://doi.org/10.21105/JOSS.03021.

65. Longtine, M.S., Mckenzie III, A., Demarini, D.J., Shah, N.G., Wach, A., Brachat, A., Philippsen, P., and Pringle, J.R. (1998). Additional modules for versatile and economical PCR-based gene deletion and modification in Saccharomyces cerevisiae. Yeast *14*, 953–961. https://doi.org/10.1002/(SICI)1097-0061(199807)14:10<953::AID-YEA293>3.0.CO;2-U.

66. Janke, C., Magiera, M.M., Rathfelder, N., Taxis, C., Reber, S., Maekawa, H., Moreno-Borchart, A., Doenges, G., Schwob, E., Schiebel, E., and Knop, M. (2004). A versatile toolbox for PCR-based tagging of yeast genes: New fluorescent proteins, more markers and promoter substitution cassettes. Yeast *21*, 947–962. https://doi.org/10.1002/yea.1142.

67. Tollervey, D. (1987). A yeast small nuclear RNA is required for normal processing of pre-ribosomal RNA. EMBO J. *6*, 4169–4175. https://doi.org/10.1002/j.1460-2075.1987.tb02763.x.

68. Dietler, N., Minder, M., Gligorovski, V., Economou, A.M., Joly, D.A.H.L., Sadeghi, A., Chan, C.H.M., Koziński, M., Weigert, M., Bitbol, A.F., and Rahi, S.J. (2020). A convolutional neural network segments yeast microscopy images with high accuracy. Nat. Commun. *11*, 5723. https://doi.org/10.1038/s41467-020-19557-4.

69. Padovani, F., Mairhörmann, B., Falter-Braun, P., Lengefeld, J., and Schmoller, K.M. (2022). Segmentation, tracking and cell cycle analysis of live-cell imaging data with Cell-ACDC. BMC Biol. *20*, 174. https://doi.org/10.1186/S12915-022-01372-6.

70. Pachitariu, M., Rariden, M., and Stringer, C. (2025). Cellpose-SAM: superhuman generalization for cellular segmentation. Preprint at bioRxiv. https://doi.org/10.1101/2025.04.28.651001.

CellPress
OPEN ACCESS

## STAR★METHODS

### KEY RESOURCES TABLE

| REAGENT or RESOURCE | SOURCE | IDENTIFIER |
|---|---|---|
| **Bacterial and virus strains** | | |
| NEB® 5-alpha competent E. coli (High Efficiency) | New England Biolabs | Cat# C2987H |
| **Chemicals, peptides, and recombinant proteins** | | |
| cOmplete™, EDTA-free protease inhibitor cocktail | Roche | Cat# 04693132001 |
| RQ1 RNase-free DNase | Promega | Cat# M6101 |
| RQ1 DNase stop solution | Promega | Cat# M199A |
| RNace-It™ ribonuclease cocktail | Agilent Technologies | Cat# 400720 |
| Abscisic acid (ABA) | TOKU-E | Cat# A153-500MG |
| Rapamycin | Sigma-Aldrich | Cat# 553210-1MG |
| Water, DEPC-treated, molecular biology grade | Sigma-Aldrich | Cat# 693520 |
| RNasin® ribonuclease inhibitor | Promega | Cat# N2115 |
| Thermo Scientific™ FastAP thermosensitive alkaline phosphatase (1 U/μL) | Thermo Fisher Scientific | Cat# 10699730 |
| Terminator 5′-phosphate-dependent exonuclease | Thermo Fisher Scientific | Cat# NC1460234 |
| Proteinase K, recombinant, PCR grade | Roche | Cat# 03508838103 |
| SuperScript IV | Thermo Fisher Scientific | Cat# 18090010 |
| SuperScript™ IV RT reaction buffer | Thermo Fisher Scientific | Cat# 18090050B |
| CircLigase ssDNA ligase | Thermo Fisher Scientific | Cat# NC9439450 |
| Invitrogen™ Novex™ TBE Gels, 6% | Thermo Fisher Scientific | Cat# EC6265BOX |
| Brilliant III ultra-fast SYBR® green qPCR master mix | Agilent Technologies | Cat# 600883 |
| Random primer mix | New England Biolabs | Cat# S1330S |
| **Critical commercial assays** | | |
| Agilent high-sensitivity DNA kit | Agilent Technologies | Cat# 5067-4626 |
| Ribo-Zero Gold rRNA Removal Epidemiology Kit | Illumina | Discontinued |
| Qubit™ RNA HS assay kit | Thermo Fisher Scientific | Cat# Q32852 |
| Agilent RNA 6000 Pico kit | Agilent Technologies | Cat# 5067-1513 RUO |
| **Deposited data** | | |
| RNA-sequencing in the NAB3-ABI strain grown in glucose-containing SC -Trp medium (i) without any additional treatments, (ii) with 1% (v/v) methanol, or supplemented with (iii) 500 μM ABA or (iv) 2 mM ABA. | This study | GEO accession number: GSE149746 |
| **Experimental models: Organisms/strains** | | |
| *Saccharomyces cerevisiae* BY4741 | Brachmann et al.[57] | N/A |
| *S. cerevisiae* HHY168 (W303 *tor1-1 fpr1Δ*) | Haruki et al.[14] | N/A |
| *S. cerevisiae* HHY168 *RPL13A-2xFKBP12 NAB3-FRB* | van Nues et al.[26] | N/A |
| *S. cerevisiae* BY4741 *RPL13A-PYL-3xHA NAB3-ABI-3xFLAG* | This study | N/A |
| *S. cerevisiae* BY4741 *RPL13A-PYL- 3xHA NAB3-ABI-3xFLAG-mNeonGreen PUS1-mCherry* | This study | N/A |
| *S. cerevisiae BY4741 RPL13A-PYL-3xHA RPN11-ABI-3xFLAG* | This study | N/A |
| *S. cerevisiae* BY4741 *RPL13A-PYL-3xHA RPN11-ABI-3xFLAG-mNeonGreen PUS1-mCherry* | This study | N/A |
| *S. cerevisiae* BY4741 *PRP22-AID::HYG* | This study | N/A |
| *S. cerevisiae* BY4741 *NAB3-AID::HYG* | This study | N/A |

(Continued on next page)

| Continued | | |
|---|---|---|
| REAGENT or RESOURCE | SOURCE | IDENTIFIER |
| **Oligonucleotides** | | |
| Primers for the tagging of *PRP22* and *RPN11* in the BY4741 strain and *NAB3* and *RPL13A* in BY4741 and HHY168, see Table S4 | This study | N/A |
| Primers for real-time PCR, see Table S4 | This study | N/A |
| Primers for RNAtag-Seq, see Table S4 | Shishkin et al.[58] | N/A |
| **Recombinant DNA** | | |
| pZTRL | Mendoza-Ochoa et al.[15] | N/A |
| pURA3-AID*-6FLAG | Mendoza-Ochoa et al.[15] | N/A |
| pFA6a-PYL-NatMX6 | Amin et al.[33] | N/A |
| pFA6a-ABI-KanMX6 | Amin et al.[33] | N/A |
| **Software and algorithms** | | |
| DESeq2 | Love et al.[45] | https://bioconductor.org/packages/release/bioc/html/DESeq2.html |
| Flexbar | Dodt et al.[59] | https://github.com/seqan/flexbar |
| FlowJo 10.8.1 | BD Biosciences | https://www.flowjo.com/ |
| ggplot2 | Wickham[60] | https://ggplot2.tidyverse.org/ |
| ImageJ | Schneider et al.[61] | https://imagej.net/ij/ |
| matplotlib | Hunter[62] | https://matplotlib.org/ |
| Novoalign | Novocraft | https://www.novocraft.com/products/novoalign/ |
| Omniplate | Montaño-Gutierrez et al.[63] | https://git.ecdf.ed.ac.uk/pswain/omniplate |
| Prism 8.0.1 | GraphPad Software | https://www.graphpad.com/ |
| pyCRAC | Webb et al.[44] | https://git.ecdf.ed.ac.uk/sgrannem/pycrac |
| seaborn | Waskom, 2021[64] | https://seaborn.pydata.org/ |
| TidyqPCR | Wallace and Haynes, 2021[52] | https://github.com/ropensci/tidyqpcr |
| **Other** | | |
| Amersham ImageQuant™ 800 system | Cytvia Life Sciences | Cat# 29399481 |
| BD LSR-Fortessa | BD Biosciences | Discontinued |
| EMCCD camera | Evolve, Photometrics | N/A |
| GFP-B, GFP/mCherry (59022) exciter | Nikon | N/A |
| Infinite F200 microplate reader | Tecan | Discontinued |
| LightCycler® 480 instrument II | Roche | Cat# 05015243001 |
| NovaSeq 6000 system | Illumina | Cat# 20012850 |
| OptoLED light source | Cairn Research | P111/002/000 |
| Perfect focus system (PFS) | Nikon | N/A |
| Qubit™ 4 fluorometer | Thermo Fisher Scientific | Cat# Q33238 |
| sCMOS camera | Prime95B, Photometrics | N/A |
| Ti-E inverted microscope | Nikon | Discontinued |
| 100 x 1.4 NA oil-immersion objective | Nikon | MRY10060 |
| 2100 bioanalyzer instrument | Agilent Technologies | Cat# G2938C |

## EXPERIMENTAL MODEL AND STUDY PARTICIPANT DETAILS

### Strains and growth conditions

*Saccharomyces cerevisiae* BY4741[57] was the parental reference used to generate all the strains in this study. A list of all strains is provided in Table S3.

PCR-based modification was employed to fuse *ABI* domains downstream of NAB3 and *RPN11*; to integrate the PYL epitope distally to *RPL13A*; and to C-terminally tag *NAB3* and *PRP22* with AID*,* as previously described.[65,66] The coding sequence of the *ABI* and *PYL* domains, as well as the AID tag, were amplified from the pFA6a-ABI-KanMX6, pFA6a-PYL-NatMX6, and pURA3-AID*-6FLAG vectors using the oligonucleotides listed in Table S4.[15,33] Additionally, strains to be treated with the AID technology

were transformed with the strain with the pZTRL plasmid, which codes for a β-estradiol-inducible version of *TIR1*. For clarity, a summary of the main features of each plasmid used for generating yeast strains is provided in Table S5.

The mCherry and mNeonGreen fusions were constructed via C-terminal tagging of the endogenous locus using a lithium acetate-based transformation protocol. Pus1 was tagged with mCherry as a nuclear marker in *NAB3-ABI* (SG1098) and *RPN11-ABI* (SG1109). The then ABI domain was then subsequently fused to an mNeonGreen to create *NAB3-ABI-mNeonGreen* (MS1178) or *RPN11-ABI-mNeonGreen* (MS1179).

Yeast cells were grown in YPDA medium or synthetic complete (SC) minimal medium lacking tryptophan (SC -Trp) containing 2% (w/v) glucose as a carbon source. Agar plates of the same medium were prepared by supplementing the media with agar to a final concentration of 1% (w/v). Liquid cultures were incubated at 30°C with shaking at 190 rpm. Cells grown on Petri dishes were incubated at 30°C in a static incubator for 48 h, then stored at 4°C. Liquid and solid media was supplemented with 500 μM or 2 mM ABA (TOKU-E, A153), 0.25 or 1 μM rapamycin (Sigma-Aldrich), 750 μM auxin and/or 10 μM β-estradiol.

All strains were pre-cultured in medium containing 2% (w/v) glucose for 24 h prior to the start of the experiment. The cells used in all experiments were harvested during the exponential growth phase, except for those of the *NAB3-FRB* strain grown after rapamycin was removed from the medium (Figure 5D). For spot-test and microplate reader assays, cells were grown in 5 mL cultures. While RNA-sequencing was performed on extracts of cells collected from 25 mL cultures, real-time quantitative PCR and flow cytometry were conducted on samples harvested from 100 mL cultures. RNA was isolated from cells that were either pelleted by centrifugation (3202 rcf, 5 min) or by vacuum filtration, snap-frozen by submersion in liquid nitrogen, and stored at −80°C.

## METHOD DETAILS

### Spot-test growth assays

Pre-cultures were diluted in 5 mL of SC -Trp to an $OD_{600}$ of 0.1 and incubated for 3 to 4 h at 30°C. Cells were then pelleted, washed with fresh medium, and resuspended in 1–3 mL of SC -Trp to reach an $OD_{600}$ of 1. The resulting batches of cells were equally divided among the number of conditions (i.e., plates containing 500 μM ABA, 2 mM ABA, 1 μM rapamycin, 750 μM auxin, and/or 10 μM β-estradiol). Spot-test series were performed following 10-fold dilutions of the preceding culture. After inoculation with the relevant strains, plates were incubated at 30°C for 2 days.

### Microplate reader growth assays

Yeast cells from a culture in the mid-logarithmic growth phase were resuspended in fresh medium to a starting $OD_{565}$ of 0.1. Two hundred μL was then transferred to three separate wells of a UV-sterilised 96-well black non-treated polystyrene microplate (Thermo Fisher Scientific) and its accompanying lid (Grenier). Triplicate controls comprising the same volume of sterile medium were also included for each examined condition. Absorbance was monitored in Infinite M200 (Tecan) microplate readers every 5–10 min at a constant temperature of 30°C while undergoing 535 s of orbital shaking at an amplitude of 6 mm between each recording time point.

Microplate reader data was analyzed using the Omniplate package.[63] The software corrected the $OD_{565}$ measurements by subtracting the absorbance signal from the wells containing media only and compensated for the non-linear relationship between $OD_{565}$ measurements and cell count at higher absorbance values. Corrected $OD_{565}$ values were plotted using Python's seaborn package.

### RNA extraction

For RNA sequencing, RNA extracts from replicate samples were generated from three different BY4741 colonies grown on different days. 5 mL SC -Trp medium cultures were incubated overnight before being used to inoculate 20 mL of SC -Trp, SC -Trp supplemented with 1% methanol, SC -Trp with 500 μM of ABA dissolved in 1% methanol, and SC -Trp containing 2 mM abscisic acid solubilised in 1% methanol. After 5 h of growth, cells were harvested at an $OD_{595}$ of approximately 0.5. RNA was purified following the established GTC-acid phenol extraction.[67]

For the RT-qPCR experiments assessing *SNR13* readthrough levels, 25 mL SC -Trp pre-cultures were used to inoculate 100 mL SC -Trp cultures at an initial $OD_{595}$ of 0.15. After two hours, when cultures were undergoing early exponential phase ($OD_{600}$ 0.3–0.4), 5 mL of culture were spun down (3202 rcf, 5 min) and snap frozen. Cells were then incubated with either ABA solubilised in 1% methanol to a final concentration of 500 μM or with rapamycin dissolved in ethanol to a final concentration of 1 μM. Five mL of culture was harvested from the treated flasks at regular intervals for up to 120 min following the addition of dimerisation agents. Subsequently, cells were collected by centrifugation (3202 rcf, 5 min) and washed twice in phosphate-buffered saline (PBS) before being resuspended in fresh SC-Trp medium lacking ABA or rapamycin. Similarly, 5 mL of culture were harvested periodically (15 min after removing ABA/rapamycin and then every 30 min after that) for up to 2 h.

### RNA sequencing

RNA integrity was verified using the total RNA 6000 Nano kit on the 2100 Bioanalyzer (Amersham). Sequencing libraries from 3 biological replicates were generated using a modified version of the RNAtag-Seq protocol.[58] One microgram of RNA from each sample was fragmented in FastAP phosphatase buffer for 6 min at 92°C in a final reaction volume of 20 μL. Subsequently, sheared RNA was dephosphorylated using 10 units of FastAP phosphatase and treated with 8 units of TURBO DNase (Thermo Fisher Scientific) in the presence of 40 units of rRNasin RNase inhibitor (Promega) for 30 min at 37°C in a 40 μL mix. Three prime adapters (Table S4;

'RNAtag_3adap') were ligated to the dephosphorylated 3′ ends for 90 min at 22°C in a 40 μL reaction volume with 1x T4 RNA ligase buffer, 9% DMSO, 10mM ATP, 20% PEG8000 and 24 units rRNAsin (Promega). RNA was phenol-chloroform extracted and purified by ethanol precipitation.

Ribosomal RNA was subsequently removed using the Ribo-Zero Gold rRNA Removal Epidemiology Kit (Illumina) according to the manufacturer's procedures. Free adapters were removed using the Terminator 5′-phosphate-dependent exonuclease (Thermo Fisher Scientific) according to the manufacturer's procedure. The cDNA synthesis reaction was performed with Superscript IV following the manufacturer's procedures at 55°C for one hour in a final volume of 14 μL. RNA was removed by adding 4 μL of 1N NaOH. A second ligation reaction was performed with the 3Tr3 DNA adapter (Table S4) with CircLigase ssDNA ligase (Thermo Fisher Scientific) according to the manufacturer's procedures. Free adapters were subsequently removed using the Terminator 5′-phosphate-dependent exonuclease. The cDNA library was amplified by PCR using the P5 forward and barcoded reverse primers (2P_rev_1 and 2P_rev_2; Table S4) for 24 cycles. PCR products were resolved on a 6% TBE gel (Thermo Fisher Scientific), from which 180–400 bp fragments were excised. Final DNA concentration was measured using the Qubit 4 (Thermo Fisher Scientific) and the high-sensitivity DNA assay on the 2100 Bioanalyzer (Agilent).

### RNA-sequencing analysis

RNA sequencing was performed at NovoGene (Cambridge; 150bp PE). Raw FASTQ files were processed using the pyCRAC package (version 1.4.6[44]) using the CRAC_pipeline_PE pipeline.[26] Briefly, adapter trimming was performed using Flexbar (version 3.4.0[59]) and reads were demultiplexed using pyBarcodeFilter.py. To remove PCR duplicates, reads were collapsed using the pyFastqDuplicateRemover.py script. Subsequently, FASTA files with collapsed reads were aligned to the yeast genome (R64-1-1) using Novoalign (novoalign -d Saccharomyces_cerevisiae.R64-1-1.75.novoindex -f forward_reads.fasta reverse_reads.fasta -r Random; version 2.04; www.novocraft.com), and read counts were generated using pyReadCounters.py. Differential analysis of gene expression was performed using the DESeq2 R package.[45]

### Real-time quantitative reverse transcription PCR

RNA extracts were purified using RQ1 RNase-Free DNase (Promega) and reverse-transcribed by SuperScript IV reverse transcriptase in combination with a random primer mix (New England Biolabs). Resulting samples were combined with Brilliant III ultra-fast SYBR green qPCR master mix (Agilent Technologies), distributed in triplicate within a 384-well plate (Roche), and amplified in a LightCycler 480 qPCR instrument (Roche). Technical replicates were run in triplicate from the same RNA and averaged prior to further analysis. *RDN5* and *PGK1* served as reference transcripts in all assays. Sequences for all RT-qPCR primers are listed in Table S4. Data were processed using the TidyqPCR package.[52]

### Cell size measurements using flow cytometry

1 mL from mid-logarithmic cultures was harvested, and isolated cells were fixed by the addition of 1 mL of cold 70% ethanol and incubated at −20°C overnight. On the following day, cells were permeabilised by two 10-min incubations with 800 μL of 50 mM sodium citrate buffer (pH 7.2) at room temperature. RNace-It ribonuclease cocktail (i.e., a mixture of RNase A and RNase T1; Agilent Technologies) was then administered to each sample at a final concentration of 0.1 mg/mL before incubating them at 37°C overnight. Following RNA digestion, proteins were hydrolyzed by adding 10 μL of 20 mg/mL proteinase K (Roche) and incubating the samples at 55°C for 2 h. After sonication (5 s, 25 μm), samples could then either be stored for up to 1 month at 4°C or stained with 50 μg/mL propidium iodide (Sigma-Aldrich) for flow cytometry. For the latter instance, 200 μL of each set of cells were transferred into individual wells of a clear 96-well plate (Thermo Fisher Scientific) and analyzed in a BD LSR-Fortessa (BD Biosciences) at the Flow Cytometry Facility for the School of Biological Sciences of the University of Edinburgh. A total of 10,000 events were acquired per sample.

### Flow cytometry analyses

Experimental files were exported in their native FCS format and analyzed in FlowJo 10.8.1. Before proceeding with downstream analyses, we introduced a gate to select for single cells. For cell size experiments, the normalised distribution of FSC-A values was plotted in histograms. The weighted average and standard deviation were calculated across the three independent biological repeats assessed per cell type and condition. Strains were compared using unpaired t-tests run in GraphPad Prism 8.1, which was also used to generate the summary bar graphs.

### Microscopy-based assessment of ABA anchor-away nuclear depletion and recovery

Cells were inoculated into SC medium containing 2% glucose and cultured for 24 h at 30°C, diluted to ensure cells were always in exponential growth ($OD_{600}$ below 0.4). *NAB3-ABI-mNG* and *RPN11-ABI-mNG* were plated on a concanavalin A-coated (5 mg/mL Concanavalin A in 50 mM $CaCl_2$ and 50 mM $MnCl_2$) μ-Slide 8 Well high Glass Bottom plate (Ibidi, IB-80807). Imaging was performed on a confocal Nikon Ti2 widefield/CSU-W1 spinning disk microscope equipped with an incubation chamber and a 100 x 1.49 NA oil immersion objective. To assess AA nuclear depletion efficiency, time series were acquired across 11 z-planes with a step size of 500 nm and imaged at 15-min time intervals. Cells were treated with either 1% methanol or 2 mM abscisic acid 14 min after the start of imaging. For the washout experiments, 73 min after adding the drug, cells were washed five times with 500 μL SCD. mCherry fluorescence was acquired in the Texas Red channel (561 nm excitation, 641/75 nm emission, 8% intensity, 400 ms exposure), and

mNeonGreen fluorescence was acquired in the GFP channel (488 nm excitation, 525/30 nm emission, 8% intensity, 400 ms exposure). Brightfield images were also collected to define cell boundaries. For the images shown in Figures S2–S5, exposure and contrast were manually adjusted in ImageJ using the same settings across the entire experiment, allowing qualitative assessment of mNeonGreen distribution within cells.

## QUANTIFICATION AND STATISTICAL ANALYSIS

All statistical details, including exact *n* values, are reported in the figure legends. For microplate reader growth assays (Figures 2C, 2D and 7C), three technical replicates individual wells) were measured per condition, and two independent clonal isolates were examined per ABA-dependent AA strain; plotted lines and shading represent the mean and SD, respectively. For RNA-seq differential expression analysis (Figure 3), sequencing libraries were prepared from three independent biological replicates, each derived from a distinct colony grown on a separate day; *n* represents independent cultures. DESeq2 was used to identify differentially expressed genes; a gene was considered differentially expressed if the absolute log2(fold-change) exceeded 1 and the false discovery rate (FDR) was $\leq$0.05. For RT-qPCR quantification of 3′-extended *SNR13* transcripts (Figures 4B–4E), $n = 3$ independent biological experiments; transcript levels were normalised to the reference genes *RDN5* and *PGK1* using the TidyqPCR[52] package and expressed relative to the value at t = 0. Bar graphs show the mean ± SD. Statistical comparisons between ABA- and rapamycin-treated conditions (Figures 4D and 4E) used a two-sided paired Student's *t* test. For flow cytometry-based cell size measurements (Figures 6B and 6C), $n = 3$ independent biological repeats per strain; *n* represents independent cultures. The weighted mean and SD of FSC-A values were calculated per strain per condition, and strains were compared using a two-sided unpaired Student's *t* test; analyses were performed in GraphPad Prism 8.1.

For quantification of nuclear-to-cytosolic mNeonGreen signal, the most in-focus Z-plane of each image was identified by manual inspection and used for segmentation. Cell boundaries were segmented automatically from brightfield images using YeaZ-v2[68] with standard settings, followed by manual correction of segmentation errors in Cell-ACDC.[69] Nuclei were segmented automatically from the Pus1-mCherry channel using Cellpose_v4[70] (diameter setting 30 nm, all other settings standard), followed by manual correction. A separate segmentation of the cytosolic compartment was then performed using the Track sub-cellular objects function with tracking mode enabled. Cells lacking corresponding nuclei, and nuclei lacking matching cells, were excluded. Pairing of cells and nuclei was validated using an intersection over area (IoA) threshold of 0.95 to ensure accurate matches. Each segmentation step generated a CSV file containing mean intensity values from the mNeonGreen channel. These values were background-subtracted and then used to calculate the nuclear-to-cytosol mNeonGreen signal ratio using custom Python scripts.

**Cell Reports Methods, Volume *6***

## Supplemental information

# ABA-AA: A simple, reversible, and non-toxic anchor-away system for effective nuclear protein depletion

**Sofia Esteban-Serna, Tove Widén, Simon Seliner, Hanne Grosemans, Iseabail Farquhar, Matthew P. Swaffer, and Sander Granneman**

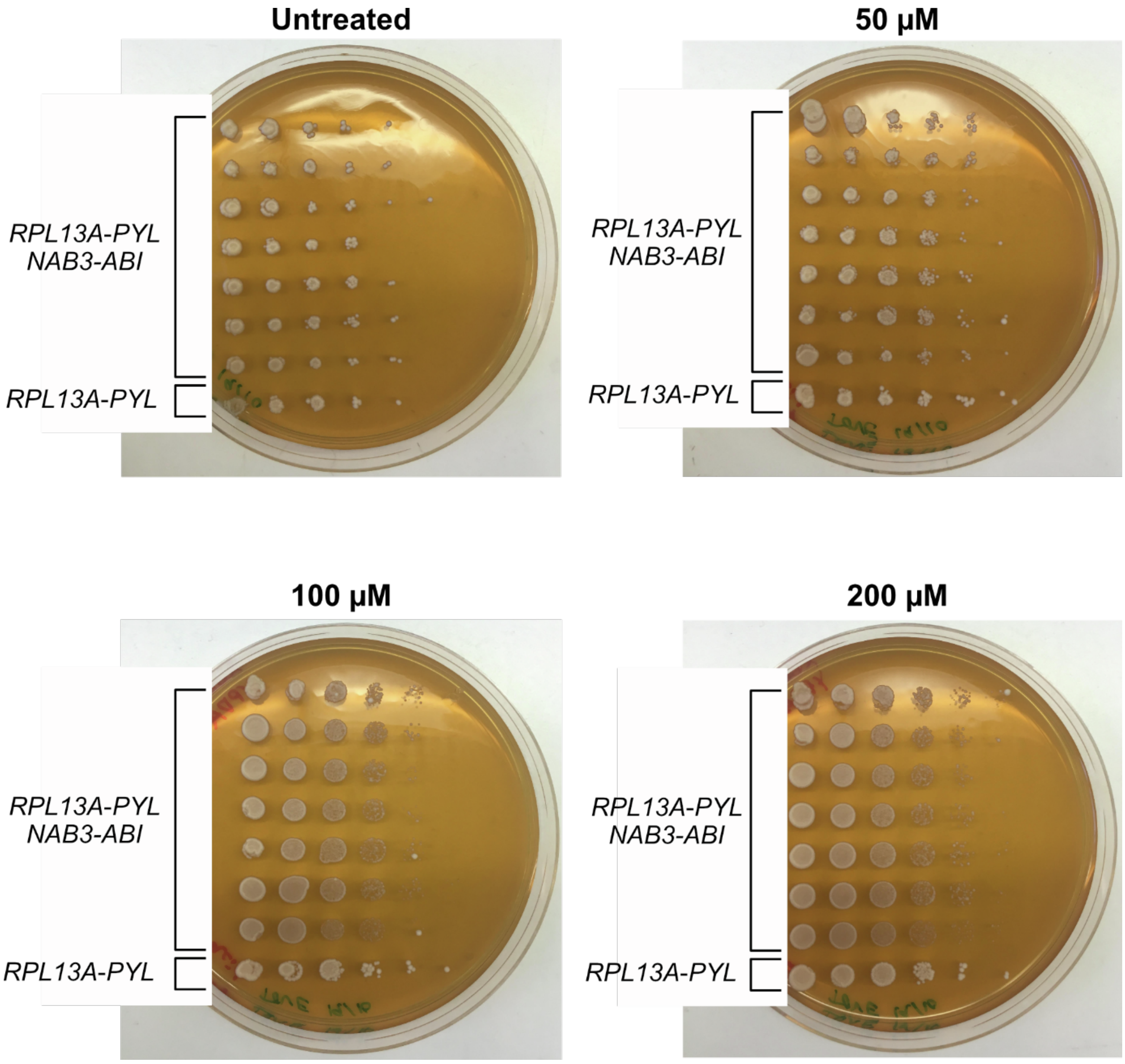


**Figure S1. ABA concentrations higher than 200 µM are required to significantly deplete Nab3 from the nucleus using ABA-AA. Related to Figure 2.**

Results from representative serial dilution growth analyses of seven *RPL13A-PYL NAB3-ABI* clones and one control strain (*RPL13A-PYL*) on YPD plates without ABA (untreated) or 50 µM, 100 µM and 200 µM ABA.

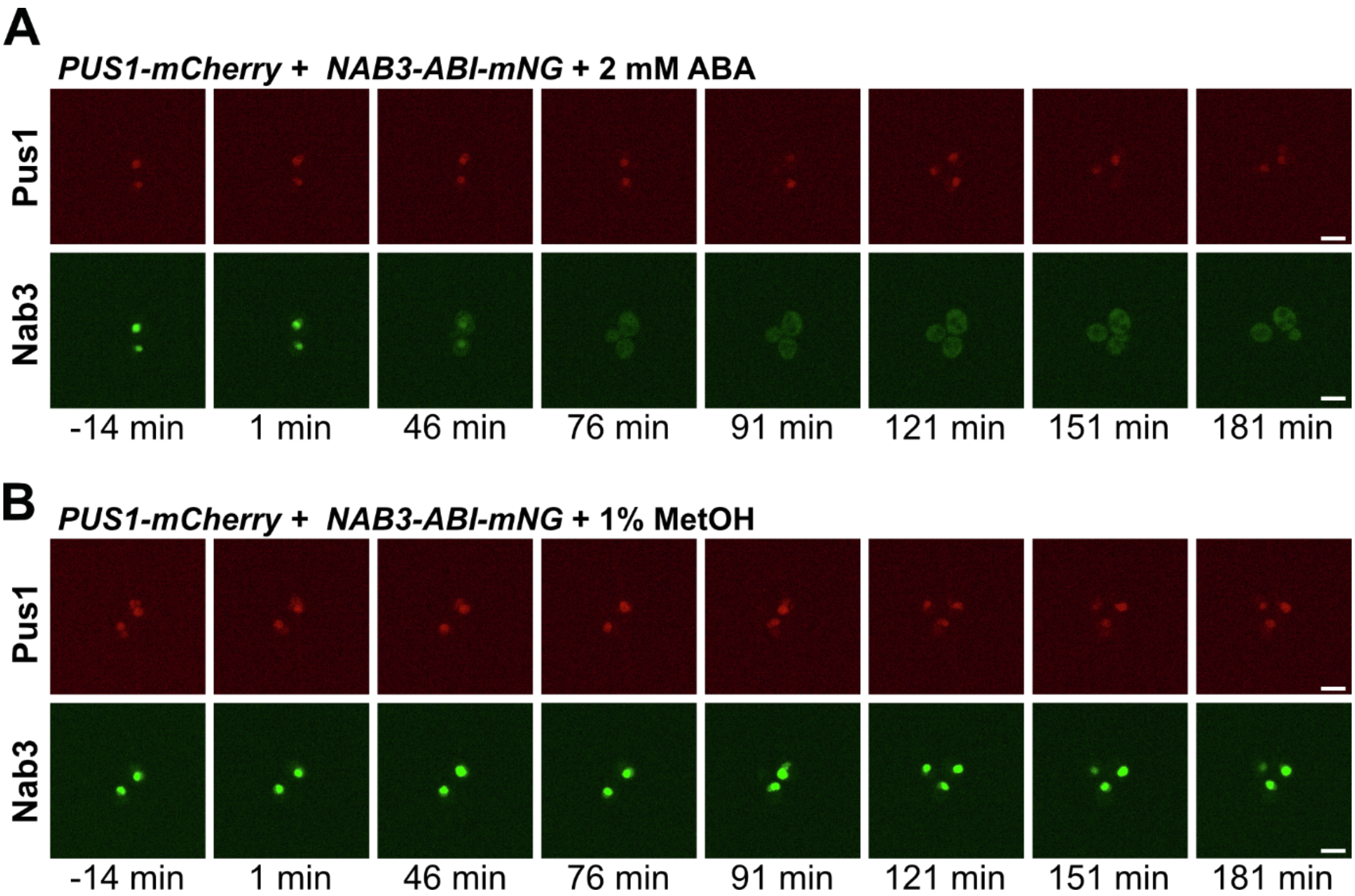


**Figure S2. ABA-AA effectively depletes Nab3 from the nucleus. Related to Figure 5.** Representative images of cells expressing Nab3-ABI-mNeonGreen. Images were acquired at the indicated time points relative to treatment with 2 mM ABA (**A**) or 1% methanol solvent (**B**). The top row shows the Pus1-mCherry signal, used as a nuclear marker. The bottom row shows the mNeonGreen signal. Quantification of the results is provided in Fig. 5A. White scale bar represents 5 µm.

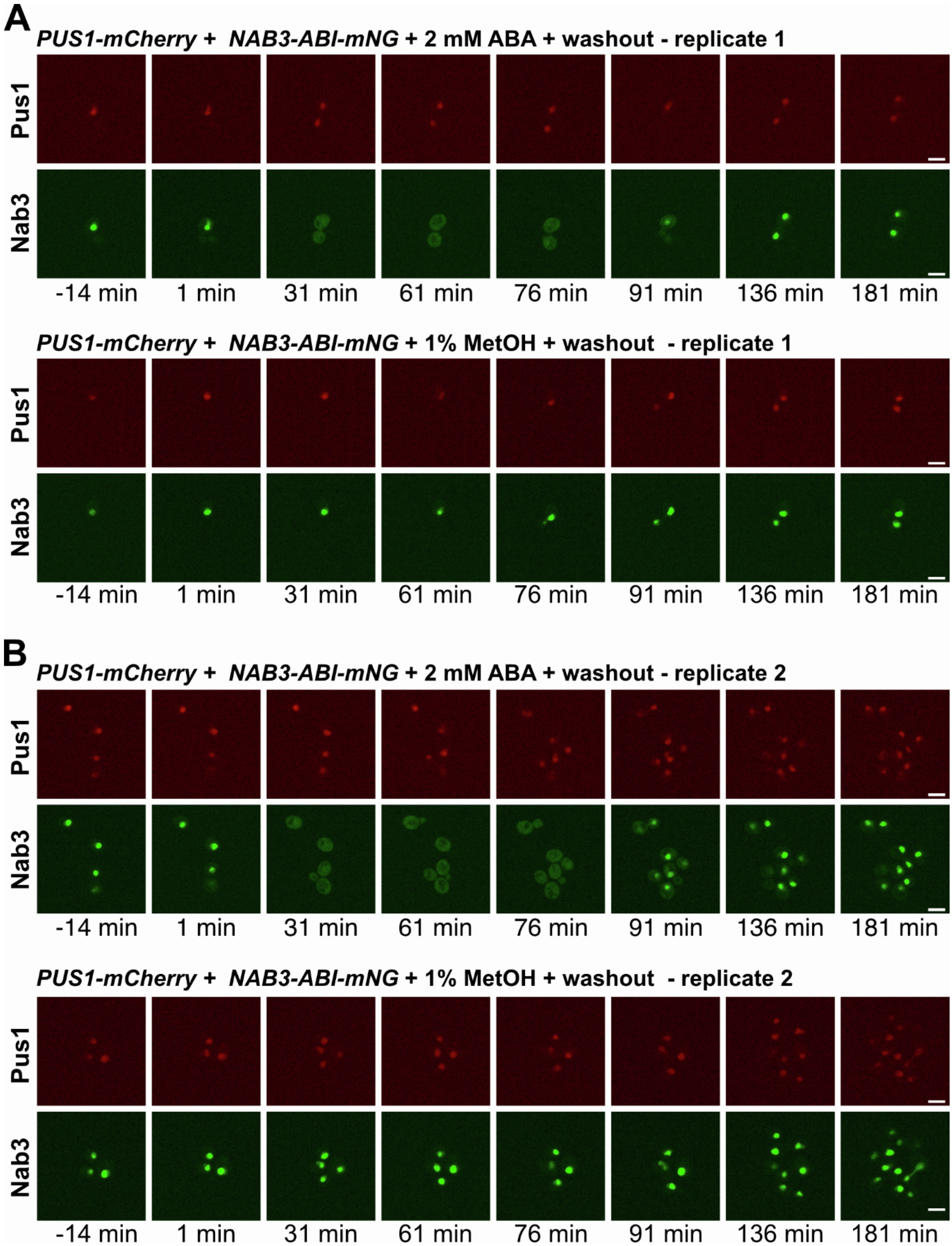


**Figure S3. Fully reversible nuclear depletion of Nab3 using ABA-AA. Related to Figure 5.** Representative images of cells expressing Nab3-ABI-mNeonGreen. Images were acquired at the indicated time points relative to initial treatment with 2 mM ABA (**A**) or 1% methanol solvent (**B**). ABA and methanol solvent were then washed out with fresh media at 73 minutes. The top row shows the Pus1-mCherry signal, used as a nuclear marker. The bottom row shows the mNeonGreen signal. Quantification of the results is provided in Fig. 5C. White scale bar represents 5 μm.

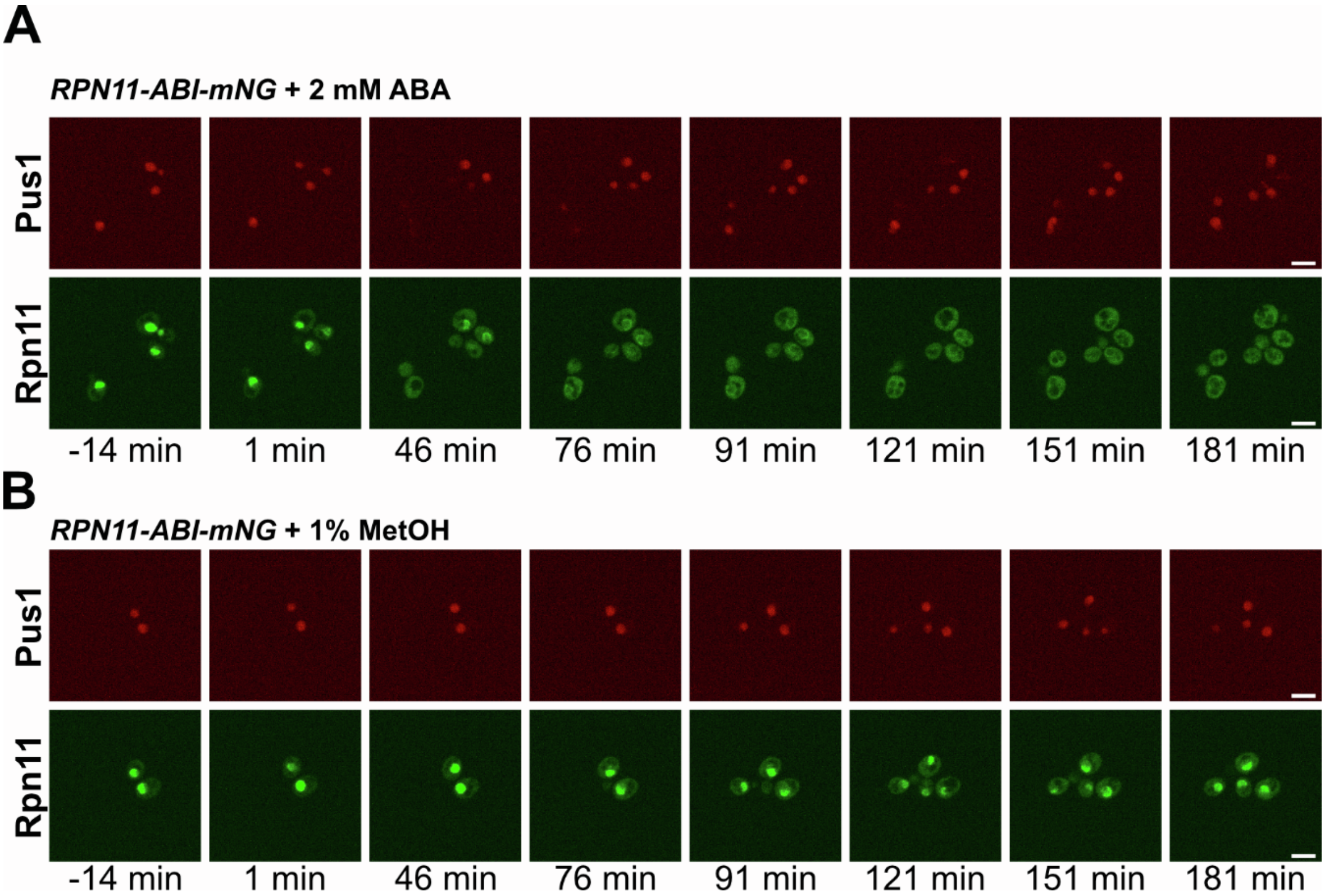


**Figure S4. ABA-AA effectively depletes Rpn11 from the nucleus. Related to Figure 5.** Representative images of cells expressing Rpn11-ABI-mNeonGreen. Images were acquired at the indicated time points relative to treatment with 2 mM ABA (**A**) or 1% methanol solvent (**B**). The top row shows the Pus1-mCherry signal, used as a nuclear marker. The bottom row shows the mNeonGreen signal. Quantification of the results is provided in Fig. 5B. White scale bar represents 5 µm.

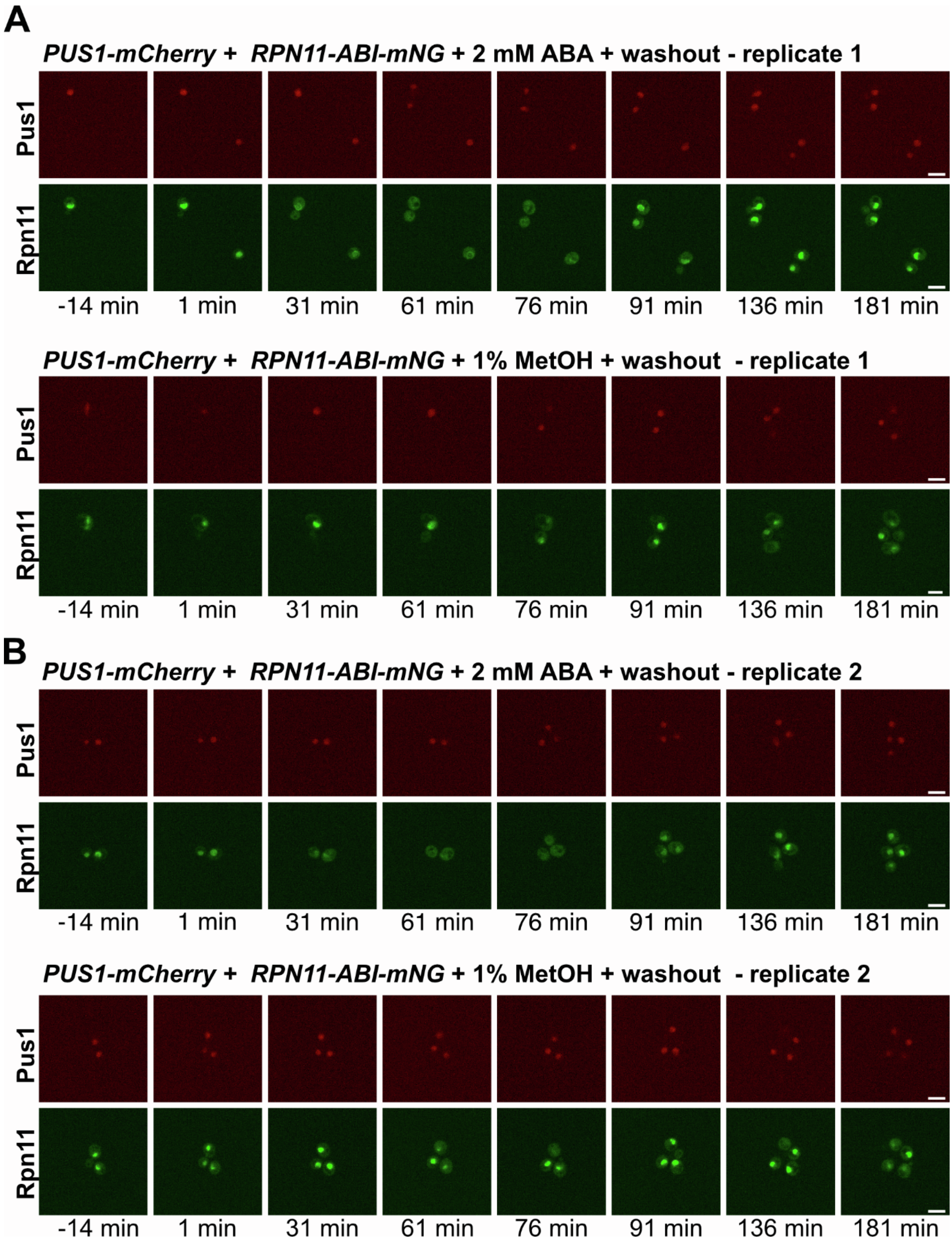


**Figure S5. Fully reversible nuclear depletion of Rpn11 using ABA-AA. Related to Figure 5.** Representative images of cells expressing Rpn11-ABI-mNeonGreen. Images were acquired at the indicated time points relative to initial treatment with 2 mM ABA (**A**) or 1% methanol solvent (**B**). ABA and methanol were then washed out with fresh media at 73 minutes. The top row shows the Pus1-mCherry signal, used as a nuclear marker. The bottom row shows the mNeonGreen signal. Quantification of the results is provided in Fig. 5D. White scale bar represents 5 μm.
